# Meta-analysis of diabetic nephropathy associated genetic variants in inflammation and angiogenesis involved in different biochemical pathways

**DOI:** 10.1186/s12881-014-0103-8

**Published:** 2014-10-04

**Authors:** Nyla Nazir, Khalid Siddiqui, Sara Al-Qasim, Dhekra Al-Naqeb

**Affiliations:** Strategic Center for Diabetes Research, King Saud University, P.O. Box 18397, Riyadh, 11415 K.S.A Saudi Arabia

**Keywords:** Diabetic nephropathy, Inflammatory cytokines, Angiogenesis, Genetic variants, Meta-analysis, Pathways, SNP

## Abstract

**Background:**

Diabetes mellitus is the most common chronic endocrine disorder, affecting an estimated population of 382 million people worldwide. It is associated with microvascular and macrovascular complications, including diabetic nephropathy (DN); primary cause of end-stage renal disease. Different inflammatory and angiogenic molecules in various pathways are important modulators in the pathogenesis and progression of diabetic nephropathy. Differential disease risk in DN may be partly attributable to genetic susceptibility. In this meta-analysis, we aimed to determine which of the previously investigated genetic variants in these pathways are significantly associated with the development of DN and to examine the functional role of these genes.

**Methods:**

A systematic search was conducted to collect and analyze all studies published till June 2013; that investigated the association between genetic variants involved in inflammatory cytokines and angiogenesis and diabetic nephropathy. Genetic variants associated with DN were selected and analyzed by using Comprehensive Meta Analysis software. Pathway analysis of the genes with variants showing significant positive association with DN was performed using Genomatix Genome Analyzer (Genomatix, Munich, Germany).

**Results:**

After the inclusion and exclusion criteria for this analysis, 34 studies were included in this meta-analysis. 11 genetic variants showed significant positive association with DN in a random-effects meta-analysis. These included genetic variants within or near *VEGFA*, *CCR5*, *CCL2*, *IL-1*, *MMP9*, *EPO*, *IL-8*, *ADIPOQ* and *IL-10*. rs1800871 (T) genetic variant in *IL-10* showed protective effect for DN. Most of these eleven genetic variants were involved in GPCR signaling and receptor binding pathways whereas four were involved in chronic kidney failure. rs833061 [OR 2.08 (95% CI 1.63-2.66)] in the *VEGFA* gene and rs3917887 [OR 2.04 (95% CI 1.64-2.54)] in the *CCL2* gene showed the most significant association with the risk of diabetic nephropathy.

**Conclusions:**

Our results indicate that 11 genetic variants within or near *VEGFA*, *CCR5*, *CCL2*, *IL-1*, *MMP9*, *EPO*, *IL-8*, *ADIPOQ* and *IL-10* showed significant positive association with diabetic nephropathy. Gene Ontology or pathway analysis showed that these genes may contribute to the pathophysiology of DN. The functional relevance of the variants and their pathways can lead to increased biological insights and development of new therapeutic targets.

**Electronic supplementary material:**

The online version of this article (doi:10.1186/s12881-014-0103-8) contains supplementary material, which is available to authorized users.

## Background

Diabetes mellitus (DM) is a set of metabolic disorders characterized by hyperglycemia resulting from defects in insulin secretion and/or action. The prevalence of the disease, which is becoming a major world-wide health problem, is increasing rapidly [1]. In 2013, 382 million cases of DM worldwide were estimated, and that number is expected to increase to 592 million cases in 2035 [1]. Diabetes mellitus is associated with microvascular and macrovascular complications, including diabetic nephropathy (DN), a primary cause of end-stage renal disease (ESRD) [2]. Due to the global increase in prevalence of diabetes there has been a concomitant rise in the number of patients with diabetic nephropathy (DN) indicating a prevalence of 30-40% of the patients with type 1 (T1DM) and type 2 diabetes (T2DM) being affected [3].

Mostly, individuals with long durations of diabetes and poor glycemic control develop progressive diabetic nephropathy. However, some patients appear to be at increased risk while others remain relatively protected [4]. Genetic predisposition plays an important role in the risk to developing diabetic nephropathy though the incidence and severity is affected by the extent of control of the abnormal metabolic state associated with diabetes mellitus [5].

In recent years, there has been an increased understanding of the genetic and molecular basis of development and progression of diabetic nephropathy. Although diabetic nephropathy is traditionally considered a nonimmune disease, recent findings indicate a significant role of immune-mediated inflammatory processes in the pathophysiology of diabetic nephropathy. These include the up-regulation of inflammatory mediators, higher urinary levels of monocyte chemoattractant protein-1 and increasing macrophage influx with the progression of diabetic nephropathy [6–8]. In streptozotocin (STZ) diabetic rats, an increase in glomeruli capillaries area was observed in comparison to normal age-matched controls, implying a pronounced angiogenic reaction to the diabetic condition [9]. Higher concentration of angiogenic markers, vascular endothelial growth factor (*VEGF*) and transforming growth factor β (*TGF β*) in plasma and urine of diabetic nephropathy patients, emphasize the role of angiogenesis [10,11]. Chen and Ziyadeh, [12] reported attenuation of diabetic albuminaria by blockade of *VEGF* signaling pathway. Further, a recent study showed anti-angiogenic and anti-inflammatory DNA vaccination ameliorates the progression of glomerular pathology in an animal model of diabetic nephropathy [13]. As inflammation and angiogenesis play a crucial role in the pathomechanism of diabetic nephropathy, many studies have investigated the association of genetic polymorphism in these pathway genes with the risk of diabetic nephropathy. However, there is a lack of consistency among the reported studies due to small sample size, limited power and sparseness of data. Therefore, the aim of this meta-analysis is to analyze all published studies that investigated the association of genetic variants involved in inflammatory cytokines and angiogenesis with diabetic nephropathy and to assess their role in different biochemical pathways.

## Methods

### Literature search strategy

The articles relevant to this study were searched from PubMed, Embase and Cochrane Library in June, 2013. Different possible variations and combinations of the following search terms were used: ‘diabetes mellitus’, ‘diabetic nephropathy’, ‘ESRD’, ‘inflammatory cytokines’, ‘angiogenesis’, ‘genetic variants’, ‘polymorphism’, ‘SNPs’ and ‘gene’. Additional search query was used by combining the names of the specific genes and genetic variants with the term ‘diabetic nephropathy’. The reference list of each relevant publication was also examined to identify additional studies appropriate for inclusion in the meta-analysis. In the initial search no filter for language preference was used.

### Inclusion and exclusion criteria

Only those studies were included in which the cases had diabetes mellitus with macroalbuminuria, overt proteinuria, ESRD due to diabetic nephropathy or diabetic nephropathy identified by biopsy and controls had diabetic mellitus with normoalbuminaria after > 10 years of diabetes duration. Cases with macroalbuminaria and/or overt proteinuria and/or ESRD were merged together for comparison with controls. The selected studies should provide detailed genotypic data of the genes in the inflammatory and angiogenic pathways. Only articles in English language were included. The cases and controls in the studies included were matched by duration of diabetes and/or age. Lack of information related to the distribution of genotypes or alleles within the case and control groups is one of our exclusion criteria. Studies in which the controls were non-diabetic and/or there was comparison of different stages of diabetic nephropathy were excluded. Review and meta-analysis articles were excluded.

### Data extraction and Statistical analysis

Following information was extracted from each selected study like PMID No, first author’s surname, journal & year of publication, sample size, ethnicity, methodology, type and duration of diabetes, criteria for diabetic nephropathy and frequency of alleles.

For each SNP, the allelic data from different studies was pooled and minor allele frequencies were calculated and compared between cases and controls. Data were entered into a database and a statistical analysis was performed using SPSS (version 17.0; SPSS, Chicago, IL). The pooled odds ratio was used to estimate the association between the genetic variants and diabetic nephropathy in this meta-analysis. The Odds Ratio (OR), at allele level and statistically significant P-values were validated for all the studies and recalculated for some of them, in order to remove any adjustments made within each study in cases where two different groups merged together, such as in the case of macroalbuminaria and ESRD groups. A p-value <0.05 was considered to be statistically significant. Deviation from Hardy–Weinberg equilibrium (HWE) was tested using the De Finetti program (http://ihg.gsf.de/cgi-bin/hw/hwa1.pl) for all the studies. The percentage of variation across studies due to heterogeneity rather than chance was estimated via Higgins I^2^ statistics using Comprehensive Meta Analysis Version 2.0 software (Biostat, Englewood NJ, 2005)^.^ I^2^ is estimated by the ratio (Q-df)/Q, where Q is the Cochran’s Q statistic and df is degrees of freedom. I^2^ lies between 0 and 100% with values over 50% indicating high heterogeneity [14]. The random-effects model was performed using Comprehensive Meta Analysis Version 2.0 software. The random effects model assumes that there is a different underlying effect size for each study. Random effects model takes into account the diversity between the studies and is preferred in majority of genetic association studies. Random effects model is generally used in the presence or anticipation of any heterogeneity between studies [15]. The sub-group analysis was performed for diabetes mellitus type (type 1 or type 2), diabetic nephropathy stage (established diabetic nephropathy or advanced diabetic nephropathy) and ethnicity (European or Asian origin). The genes showing association with diabetic nephropathy were used as input core data for Genomatix Pathway Analysis (Genomatix, Munich, Germany).

## Results

The initial literature search yielded 751 citations with 111 involving inflammatory cytokines and angiogenesis related to diabetic nephropathy in humans. 25 research articles representing 34 different studies were selected after exclusion of studies which included progression of nephropathy, meta-analysis, reviews etc. The selected 34 studies contained 55 genetic variants in 18 genes of inflammatory cytokines and angiogenesis which were associated with diabetic nephropathy. Table 1 includes the details and references of all the studies used in this meta-analysis. The detailed information of the studied SNPs and the corresponding pooled odds ratios and p-values are presented in Additional file 1a, b, c, d and e.

**Table 1:** **Details of the genes and studies in this meta-analysis study**

**Gene**	**SNP**	**Article**	**No. of studies**	**Population**	**Case definition**	**T1D/T2D**	**OR** ^**a**^
CCR5 (Chemokine Receptor 5)	rs7637813	Pettigrew et al, [16]	1	Irish	EDN^b^	1	1.173(0.932-1.478)
	10577983	Pettigrew et al, [16]	1				1.163(0.935-1.447)
	rs2227010	Pettigrew et al, [16]	1				1.0(0.805-1.248)
	rs17765882	Pettigrew et al, [16]	1				1.501(0.980-2.298)
	rs2734648	Tregouet et al, [17]	3	Danish	EDN	1	0.969(0.788-1.191)
		Tregouet et al, [17]		Finnish	EDN	1	1.186(0.947-1.486)
		Tregouet et al, [17]		French	EDN	1	1.082(0.853-1.373)
	Del 32/rs333	Ahluwalia et al, [18]	4	North Indian	EDN	2	2.58 (1.98–3.37)
		Ahluwalia et al, [18]		South Indian	EDN	2	0.88 (0.51–1.5)
		Pettigrew et al, [16]		Irish	EDN	1	1.242(0.859-1.794)
		Mlynarski et al, [19]		American	ADN^c^	1	0.959(0 .652 1.410)
	59029 G.A/rs1799987	Pettigrew et al, [16]	10	Irish	EDN	1	1.017(0.818-1.263)
		Ahluwalia et al, [18]		North Indian	EDN	2	2.22 (1.71–2.87)
		Ahluwalia et al, [18]		South Indian	EDN	2	2.17 (1.43–3.29)
		Buraczynska et al, [20]		Polish	EDN	2	1.83(1.43-2.34)
		Mlynarski et al, [19]		American	ADN	1	0.904(0.664- 1.23)
		Nakajima et al, [21]		Japanese	EDN	2	1.179(0.855-1.627)
		Tregouet et al, [17]		Danish	EDN	1	1.112(0.918-1.348)
		Tregouet et al, [17]		Finnish	EDN	1	1.152(0.938-1.415)
		Tregouet et al, [17]		French	EDN	1	1.063(0.845-1.336)
		Prasad et al, [22]		Indian	ADN	2	1.379(1.047-1.818)
ADIPOQ (Adiponectin, C1Q And Collagen Domain Containing)	rs266729	Zhang et al, [23]	2	European	EDN + ADN	1	1.120(0.929-1.35)
		Wu et al, [24]		Taiwanese	EDN	2	1.39(1.0-1.9)
	rs17300539	Vionnet et al, [25]	4	Danish	EDN and Ret^d^	1	1.348(0.965-1.884)
		Vionnet et al, [25]		Finnish	EDN and Ret	1	1(0.570-1.754)
		Vionnet et al, [25]		French	EDN and Ret	1	1.472(0.998-2.171)
		Prior et al, [26]		British	EDN and Ret	1	1.924 (1.024-3.617)
IL8 (Interleukin-8)	rs4073	Ahluwalia et al, [18]	2	North Indian	EDN	2	1.44 (1.1–1.88)
		Ahluwalia et al, [18]		South Indian	EDN	2	1.5 (0.96–2.33)
CCL2 (Chemokine ligand 2)	rs1024611	Ahluwalia et al, [18]	2	North Indian	EDN	2	1.04 (0.8–1.36)
		Joo et al, [27]		Korean	ADN	2	0.91(0.65-1.2)
	rs3917887	Ahluwalia et al, [18]	2	North Indian	EDN	2	2.03 (1.57–2.63)
		Ahluwalia et al, [18]		South Indian	EDN	2	1.7 (1.12–2.57)
MMP9 (Matrix Metallopeptidase 9)	rs17576	Ahluwalia et al, [18]	2	North Indian	EDN	2	1.81 (1.40–2.34)
		Ahluwalia et al, [18]		South Indian	EDN	2	2.19 (2.45–3.31)
IL-10 (Interleukin-10)	–592C/A/rs1800872	Arababadi et al, [28]	2	Iranian	EDN	2	1.484(0.944-2.331)
		Ezzidi et al, [2]/ Mtiraoui et al, [29]		Tunisian	EDN	2	0.915(0.751-1.114)
	–819C/T/rs1800871	Ezzidi et al, [2]/ Mtiraoui et al, [29]	1				0.777(0.631-0.958)
	–1082G/A/rs1800896	Ezzidi et al, [2]/ Mtiraoui et al, [29]	1				0.841(0.694-1.018)
IL-1 (Interleukin-1)	IL1A	Loughrey et al, [30]	1	Caucasian	EDN	1	0.66 (0.42-1.04)
	IL1B	Loughrey et al, [30]	1				1.971(1.221-3.182)
	IL1RI	Loughrey et al, [30]	1				0.94(0.618-1.429)
VEGFA (Vascular endothelial growth factor A)	- 2549 I/D/rs35569394	Yang et al, [31]	2	British	EDN and Ret	1	1.619(1.038-2.525)
		Buraczynska et al, [32]		Polish	EDN	2	1.09(0.77-1.54)
	+405/rs2010963	Buraczynska et al, [32]	2	Polish	EDN	2	0.949(0.658-1.369)
		McKnight et al, [33]		Irish	EDN and Ret	1	0.88 (0.69-1.12)
	-1499C > T/rs833061	McKnight et al, [33]	2	Irish	EDN and Ret	1	2.08(1.63-2.66)
	-2578C > A/rs699947	McKnight et al, [33]	2	Irish	EDN and Ret	1	0.92(0.72-1.17)
	rs2146323	Tregouet et al, [17]	3	Danish	EDN	1	0.899(0.732-1.105)
		Tregouet et al, [17]		Finnish	EDN	1	0.914(0.739-1.131)
		Tregouet et al, [17]		French	EDN	1	0.854(0.667-1.095)
	rs3024997	Tregouet et al, [17]	3	Danish	EDN	1	0.967(0.786-1.191)
		Tregouet et al, [17]		Finnish	EDN	1	0.986(0.773-1.257)
		Tregouet et al, [17]		French	EDN	1	1.124(0.879-1.438)
	rs3025000	Tregouet et al, [17]	3	Danish	EDN	1	0.962(0.781-1.186)
		Tregouet et al, [17]		Finnish	EDN	1	0.974(0.765-1.240)
		Tregouet et al, [17]		French	EDN	1	1.087(0.843-1.402)
	936C/T/rs3025039	Kim et al, [34]	2	korean	EDN + ADN	2	1.632(0.984-2.708)
VEGFB (Vascular endothelial growth factor B)	rs12366035	Tregouet et al, [17]	3	Danish	EDN	1	0.784(0.633-0.971)
		Tregouet et al, [17]		Finnish	EDN	1	0.92(0.727-1.163)
		Tregouet et al, [17]		French	EDN	1	1.144(0.887-1.474)
VEGFC (Vascular endothelial growth factor C)	rs585706	Tregouet et al, [17]	3	Danish	EDN	1	0.8(0.562-1.139)
		Tregouet et al, [17]		Finnish	EDN	1	1.318(0.917-1.895)
		Tregouet et al, [17]		French	EDN	1	1.271(0.882-1.832)
IL-6 (Interleukin-6)	rs2069827	Ng et al, [35]	1	Caucasian	EDN + ADN	2	0.65(0.365-1.159)
	rs1800796	Ng et al, [35]					0.935(0.588-1.487)
	rs1800795	Ng et al, [35]					0.829(0.623-1.104)
	rs2069837	Ng et al, [35]					0.865(0.54-1.384)
	rs2069840	Ng et al, [35]					0.916(0.686-1.225)
	rs2069861	Ng et al, [35]					1.505(0.882-2.569)
TGF-B1 (Transforming growth factor beta 1)	rs1800470	Jahromi et al, [36]	8	Caucasian	EDN	1	1.06(0.64-1.7)
		Ahluwalia et al, [18]		North Indian	EDN	2	1.1 (0.83–1.44)
		Ng et al, [37]		Caucasian	EDN + ADN	1	0.923(0.722–1.180)
		McKnight et al, [33]		Irish	EDN	1	1.2(0.7-2.1)
		Tregouet et al, [17]		Danish	EDN	1	0.979(0.799-1.199)
		Tregouet et al, [17]		Finnish	EDN	1	1.068(0.852-1.338)
		Tregouet et al, [17]		French	EDN	1	1.034(0.816-1.311)
		Salgado et al, [38]		Mexican	EDN + ADN	2	1.30(0.99-1.71)
	Tyr81His/rs111033611	Ahluwalia et al, [18]	1	North Indian	EDN	2	1.14 (0.53–2.46)
	915 G > C/rs1800471	Ng et al, [37]	3	Caucasian	EDN + ADN	2	1.022(0.627–1.665)
		McKnight et al, [33]		Irish	EDN	1	1.0(0.66-1.5)
		Salgado et al, [38]		Mexican	EDN + ADN	2	4.17(1.40-12.3)
	-800 A > Grs1800468	Ng et al, [37]	4	Caucasian	EDN + ADN	2	1.178(0.762–1.821)
		McKnight et al, [33]		Irish	EDN	1	0.75(0.45-1.2)
		Prasad et al, [22]		Indian	ADN	2	1.04(0.816-1.325)
		Salgado et al, [38]		Mexican	EDN + ADN	2	0.98(0.46-2.09)
	rs1800469	Ng et al, [37]	3	Caucasian	EDN + ADN	2	1.088(0.841–1.407)
		McKnight et al, [33]		Irish	EDN	1	0.94(0.58-1.5)
		Prasad et al, [22]		Indian	ADN	2	0.738(0.50-1.091)
	rs1800472	Ng et al, [37]	1	Caucasian	EDN + ADN	2	1.168(0.639–2.135)
	rs2241717	Tregouet et al, [17]	3	Danish	EDN	1	0.957(0.787-1.164)
		Tregouet et al, [17]		Finnish	EDN	1	1.068(0.864-1.322)
		Tregouet et al, [17]		French	EDN	1	0.917(0.729-1.155)
	rs8179181	Tregouet et al, [17]	3	Danish	EDN	1	1.193(0.957-1.488)
		Tregouet et al, [17]		Finnish	EDN	1	1.022(0.799-1.308)
		Tregouet et al, [17]		French	EDN	1	0.944(0.722-1.234)
TGF-BR1 (TGF beta receptor 1)	rs1571589	Tregouet et al, [17]	3	Danish	EDN	1	0.951(0.746-1.213)
		Tregouet et al, [17]		Finnish	EDN	1	1.111(0.836-1.476)
		Tregouet et al, [17]		French	EDN	1	1.018(0.766-1.352)
	rs928180	Tregouet et al, [17]	3	Danish	EDN	1	1.362(0.988-1.877)
		Tregouet et al, [17]		Finnish	EDN	1	0.92(0.67-1.263)
		Tregouet et al, [17]		French	EDN	1	0.901(0.608-1.334)
TGF-BR2 (TGF beta receptor 2)	747C > G/rs11466531	McKnight et al, [33]	1	Irish	EDN	1	1.37(1.0-1.89)
	1149G > A/ss50394788	McKnight et al, [33]	1	Irish	EDN	1	2.432(0.81-7.303)
CCR2 (chemokine receptor type 2)	G46295A/rs1799864	Joo et al, [27]		korean	ADN	2	0.91(0.65-1.2)
	rs1799865	Prasad et al, [22]	1	Indian	ADN	2	1.551(0.901-2.671)
RANTES/CCL5 (Chemokine ligand 5)	C-28G/rs2280788	Joo et al, [27]	2	korean	ADN	2	0.97(0.65-1.4)
		Nakajima et al, [21]		Japanese	EDN	2	1.532(1.004-2.338)
	ss161639200	Pettigrew et al, [16]	1	irish	EDN	1	1.140(0.821-1.584)
	rs9898132	Pettigrew et al, [16]	1	Irish	EDN	1	1.092(0.746-1.599)
	G-403A/rs2107538	Joo et al, [27]	3	Korean	ADN	2	0.8(0.59-1.0)
		Pettigrew et al, [16]		Irish	EDN	1	1.085(0.81-1.452)
		Nakajima et al, [21]		Japanese	EDN	2	0.88(0.632-1.225)
EPO (Erythropoietin)	rs1617640	Tong et al, [39]	3	American	EDN + ADN	2	1.446(1.145-1.826)
		Tong et al, [39]				1	1.535(1.320-1.787)
		Tong et al, [39]				1	1.382(1.050-1.820)
TNFα (Tumor necrosis factor)	-308/rs1800629	Prasad et al, [22]	1	Indian	ADN	2	1.383(0.775-2.468)

^a^OR, Odds ratio (95% CI); ^b^EDN, established diabetic nephropathy; ^c^ADN, advanced diabetic nephropathy; ^d^Ret, retinopathy.

This meta- analysis included 55 SNPs in 18 genes of inflammatory cytokines and angiogenesis which were associated with diabetic nephropathy.

**Figure 1:**
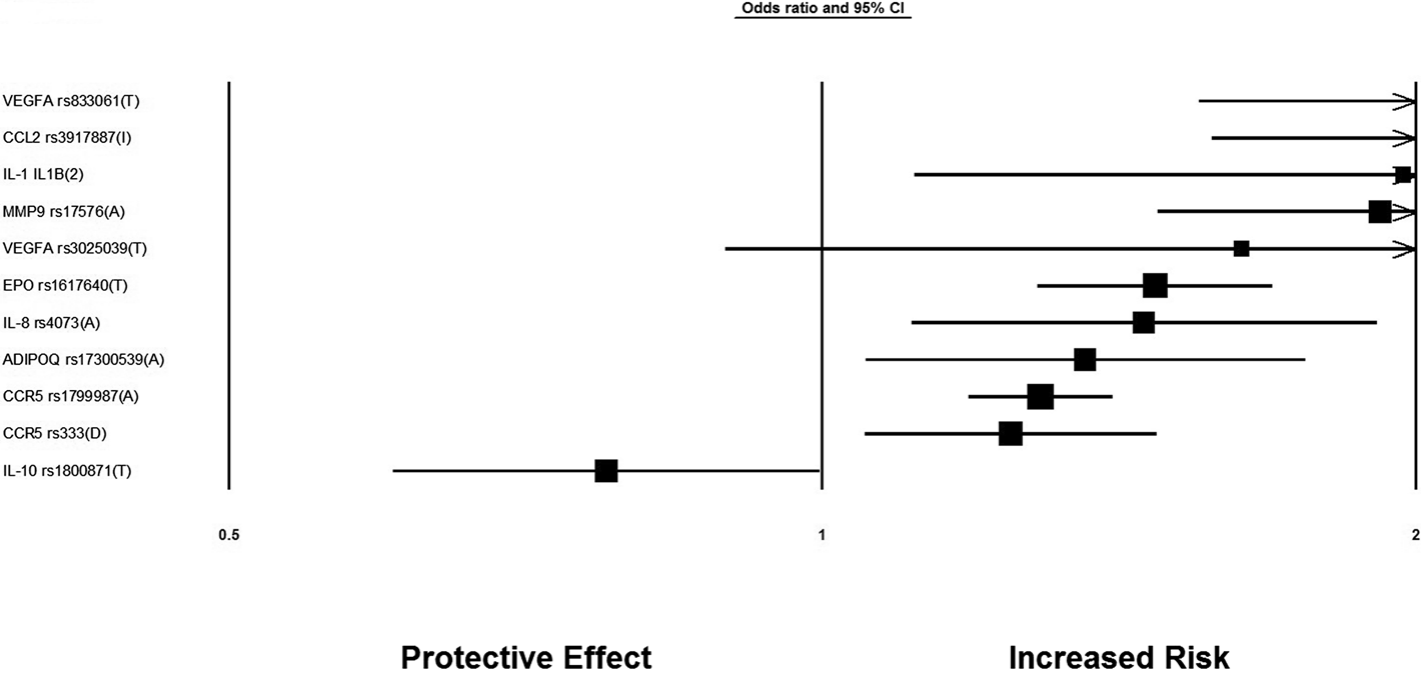
**Forest Plot for genetic variants involved in inflammatory cytokines and angiogenesis and significantly associated with diabetic nephropathy after meta-analysis.**

**Figure 2:**
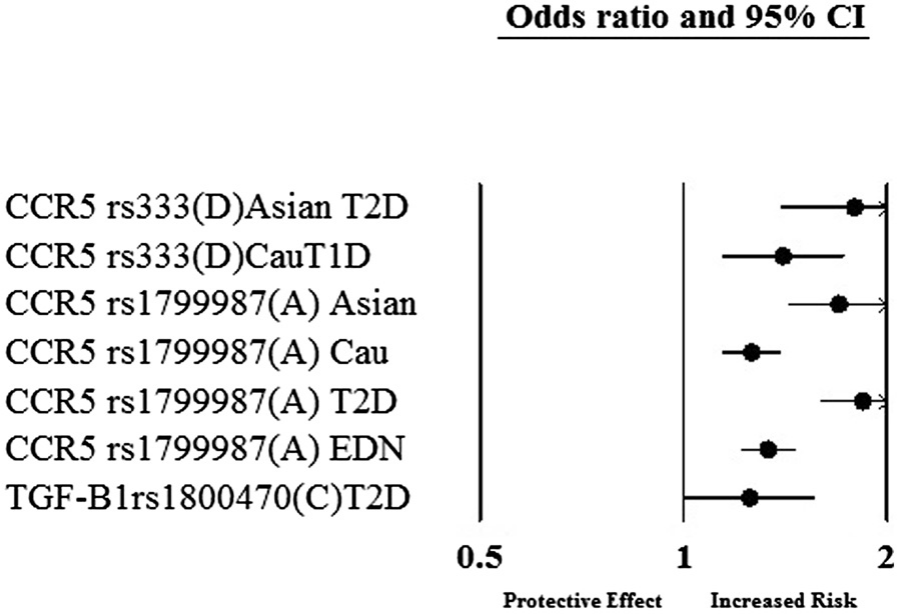
**Genetic variants significantly associated with diabetic nephropathy after meta-analysis in a subgroup; T1D- type 1 diabetes mellitus, T2D- type 2 diabetes mellitus, Cau-Caucasian origin, EDN- established diabetic nephropathy.**

**Figure 3:**
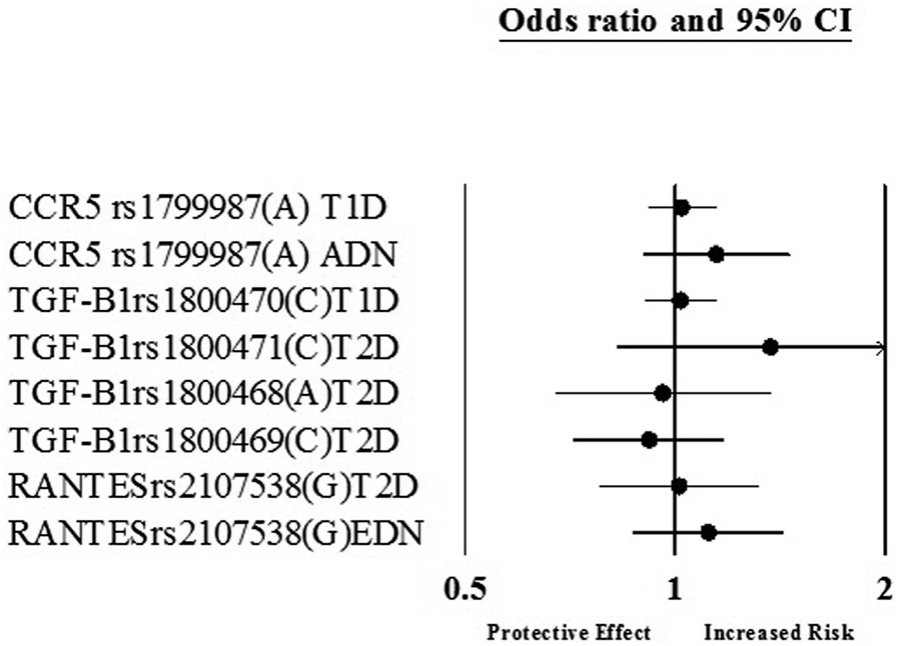
**Genetic variants not significantly associated with diabetic nephropathy after meta-analysis in a subgroup; T1D- type 1 diabetes mellitus, T2D- type 2 diabetes mellitus, EDN- established diabetic nephropathy, ADN- advanced diabetic nephropathy.**

Of the 55 genetic variants involved in the inflammatory cytokines and angiogenesis, 11 genetic variants in or near 9 genes were significantly associated with diabetic nephropathy after random-effects meta-analysis. Figure 1 is a Forest plot representation of the significantly associated genetic variants with diabetic nephropathy- *VEGFA*, *CCR5* (*Chemokine Receptor 5*), *CCL2* (*Chemokine ligand 2*), *IL-1* (*Interleukin-1*), *MMP9* (*Matrix Metallopeptidase 9*), *EPO* (*Erythropoietin*), *IL-8* (*Interleukin-8*), *ADIPOQ* (*Adiponectin, C1Q And Collagen Domain Containing*) and *IL-10* (*Interleukin-1*). The odds ratios of the significant associations with diabetic nephropathy were between the range of 1.24 to 2.08 for increased risk effect. After meta-analysis, 2 genetic variants- rs833061 in the *VEGFA* gene and rs3917887 in the *CCL2* gene showed the most significant association with risk of diabetic nephropathy. Genetic variant rs833061 was analyzed in 2 Irish studies resulting in a pooled odds ratio of 2.08 (95% CI 1.63-2.66). For rs3917887, the pooled OR was 2.04 (95% CI 1.64-2.54) obtained from 2 Indian studies. rs833061 showed significant association in type 2 diabetes mellitus whereas rs3917887 was studied in type 1 diabetes mellitus. Genetic variant rs1799987 in *CCR5* was the most studied SNP with ten studies of which six were of caucasian and four Asian. Five studies involved type 1 diabetes mellitus while the other five included type 2 diabetes mellitus resulting in a pooled odds ratio of 1.29 (95% CI 1.20-1.38). Figures 2 and 3 show the association of variants with diabetic nephropathy among sub-groups. The significant association between rs1799987 and diabetic nephropathy was reproduced in type 2 diabetes mellitus, Asian, Caucasian and established diabetic nephropathy subgroups. This association was not significant in the type 1 diabetes mellitus and advanced diabetic nephropathy subgroups. Other significantly associated genetic variant, rs333 (Del32) in the *CCR5* gene had a pooled odds ratio of 1.24 (95% CI 1.08-1.43) from four studies. Among these four studies, two Asian studies involved type 2 diabetes mellitus whereas two Caucasian studies were for type 1 diabetes mellitus. The association was reproduced in the subgroup analysis (Figure 2).

The genetic variant rs17300539 in *ADIPOQ* gene was studied in four european studies, all involving type 1 diabetes mellitus. rs17300539 was significantly associated with diabetic nephropathy with a pooled odds ratio of 1.35 (95% CI 1.09-1.68). rs4073 SNP in IL-8 gene had a pooled OR of 1.45 (95% CI 1.16-1.82) from two Indian studies involving type 2 diabetes mellitus. In the same Indian studies, another genetic variant rs17576 in the *MMP9* gene was significantly associated with diabetic nephropathy with a pooled OR of 1.91 (95% CI 1.54-2.38). The genetic variant rs3025039 in the *VEGF* gene was found to be significantly associated with diabetic nephropathy with a pooled OR of 1.63 (95% CI 0.98-2.70). Two studies in the Korean population investigated rs3025039 in type 2 diabetic patients. The genetic variant rs1617640 in the *EPO* gene was investigated in three American studies in which one study included type 2 diabetes mellitus whereas other two were for type 1 diabetes mellitus. After meta-analysis, the pooled OR of 1.47 (95% CI 1.31-1.65) showed a significant association with diabetic nephropathy. There was only one study in type 1 diabetes Caucasian population for the SNP IL1B in the *IL-1* gene. IL1B showed a significant association with risk of diabetic nephropathy having an OR of 1.97 (95% CI 1.22-3.18).

**Figure 4:**
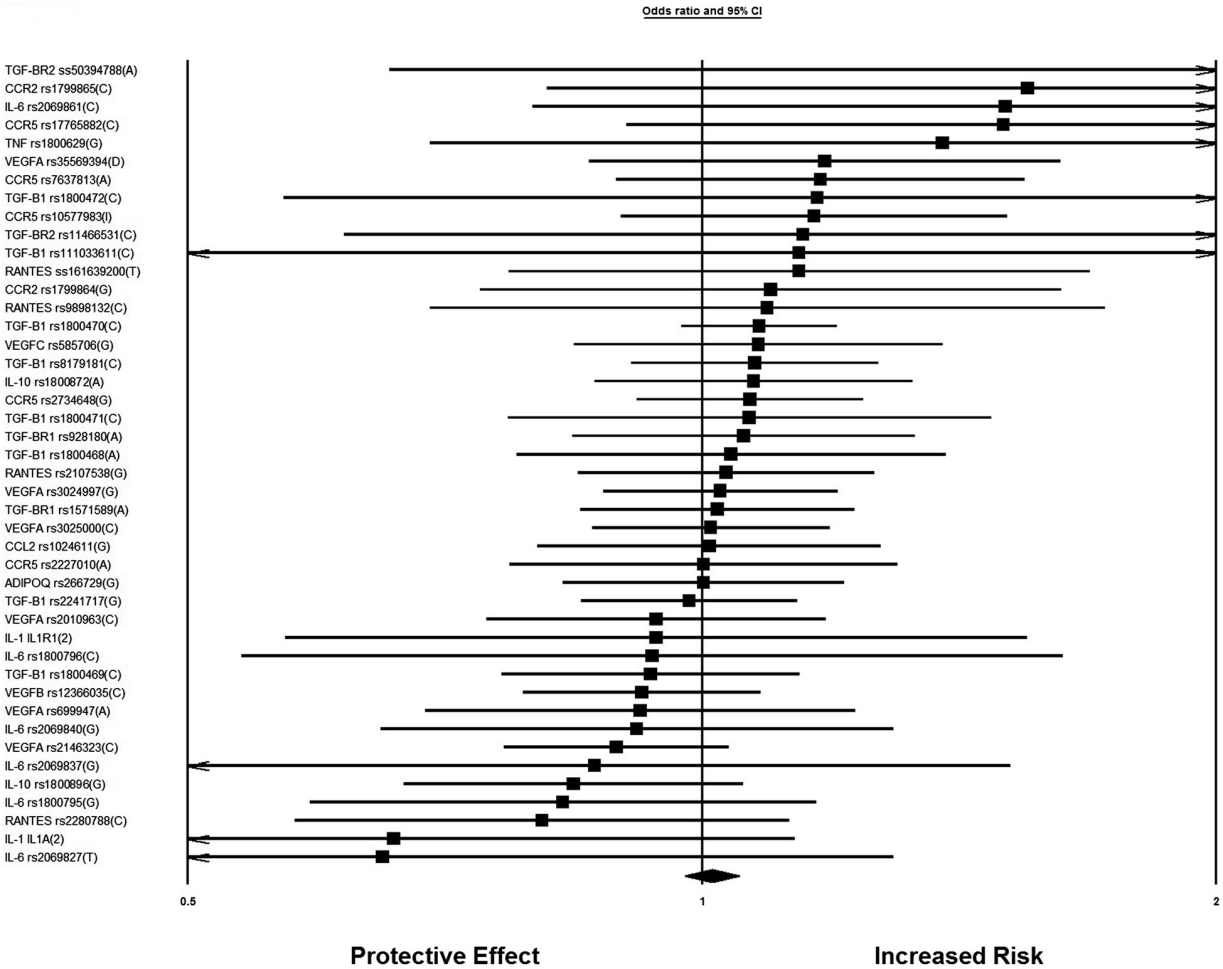
**Forest Plot for genetic variants involved in inflammatory cytokines and angiogenesis and not significantly associated with diabetic nephropathy after meta-analysis.**

In this meta-analysis, there was only one SNP which had a protective effect against diabetic nephropathy. Genetic variant rs1800871 in the *IL-10* gene was studied in a Tunisian study and had an OR of 0.77 (95% CI 0.63-0.98). In our meta-analysis, there were 44 genetic variants in the genes involved in inflammatory cytokines and angiogenesis that were not found to be significantly associated with DN (Figure 4). It is important to mention that among the 44 SNPs, only 22 were investigated in two or more than two studies while the others had a single study each. Genetic variant rs1800470 showed a significant association with diabetic nephropathy in the type 2 diabetes mellitus subgroup with an OR of 1.25 (95% CI 1.04-1.51) (Figure 2).

**Figure 5:**
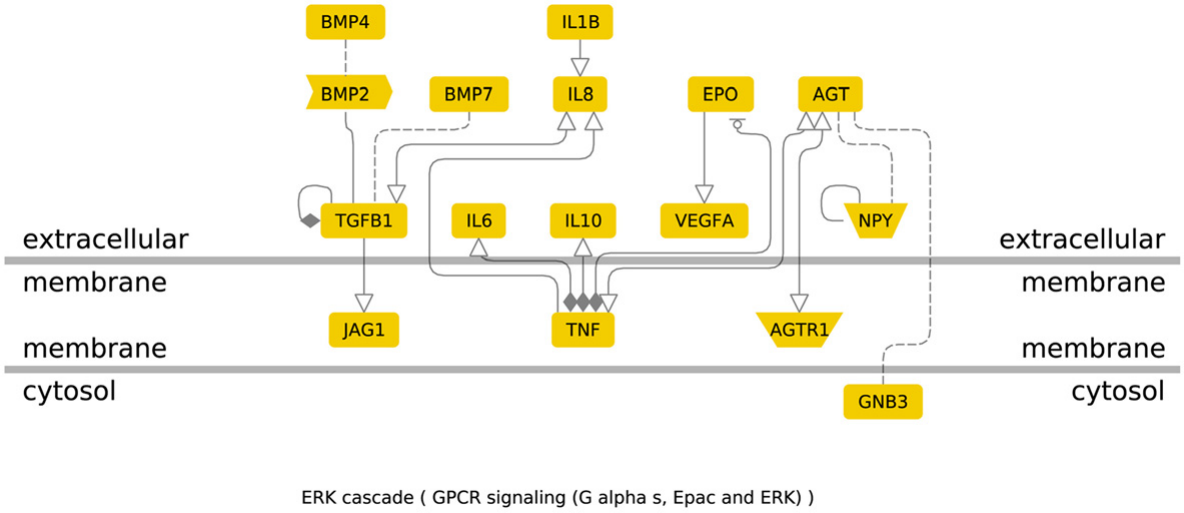
**Genes involved in GPCR signaling Pathway: (G protein coupled-receptors are seven-transmemebrane receptors involved in signal transduction in humans).**

**Figure 6:**
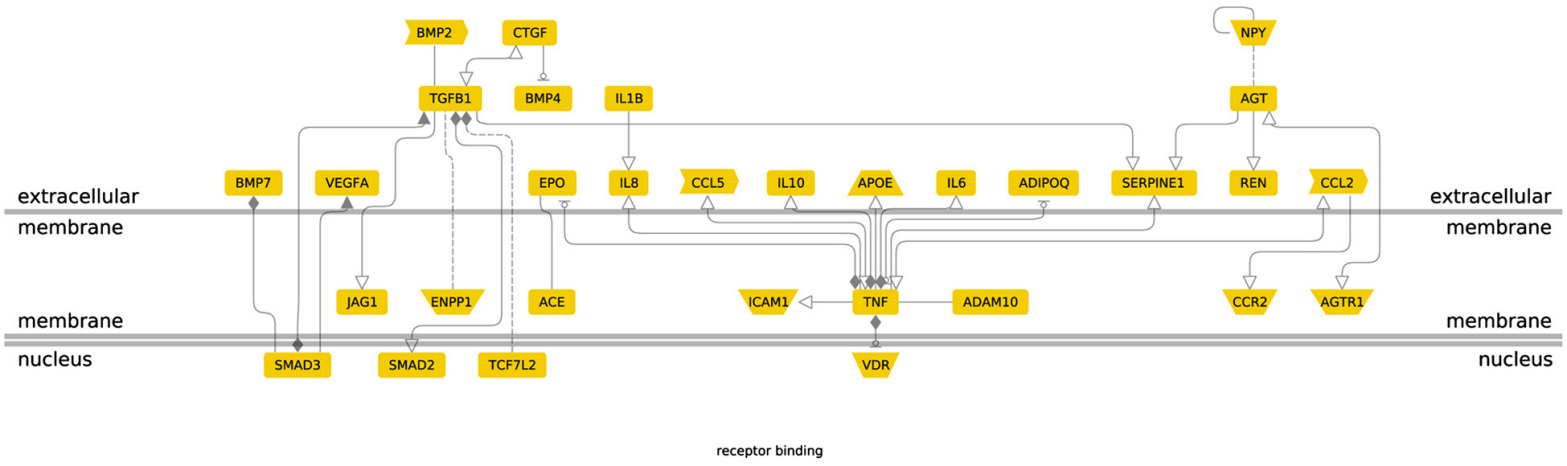
**Genes involved in receptor binding pathway.**

**Figure 7:**
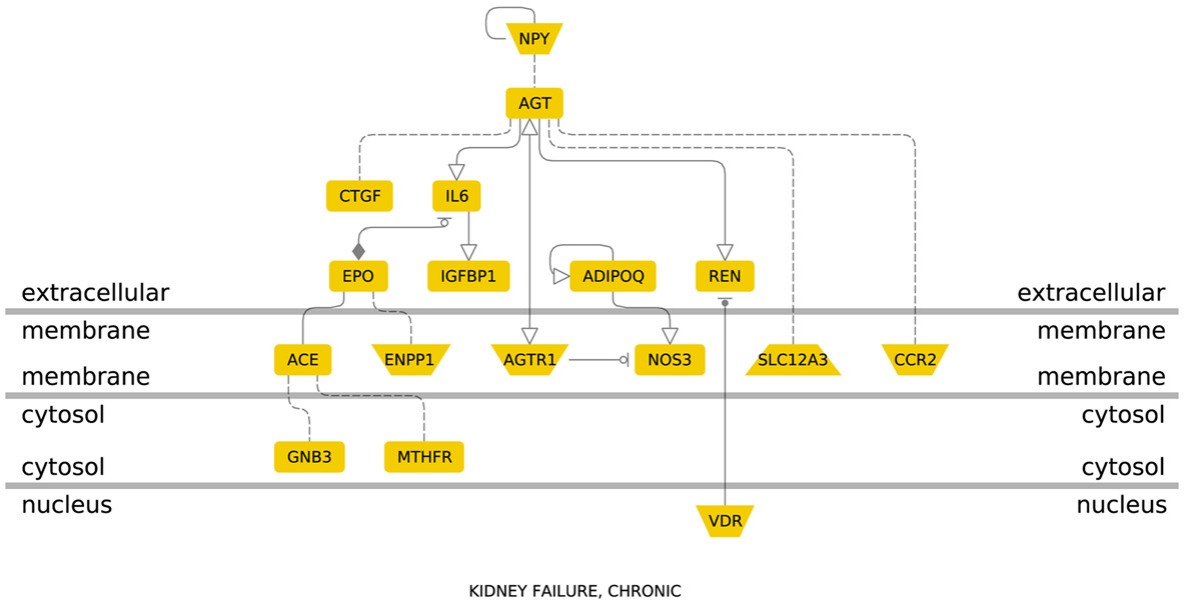
**Genes involved in chronic kidney failure (Disease Pathway).**

For this meta-analysis, genes showing significant association with diabetic nephropathy were analyzed to assess their contribution in different functional pathways. G protein coupled-receptors (GPCRs) or seven-transmemebrane receptors are the most prevailing class of signaling transduction molecules in humans. In the GPCR signaling pathways, inflammatory cytokine and angiogenic genes positively associated with diabetic nephropathy encode proteins present on the extra-cellular membrane. Among the nine genes showing significant association with diabetic nephropathy in our study- *IL10*, *VEGFA*, *EPO*, *IL1* and *IL8* were a part of GPCR signaling pathway (Figure 5). In the molecular function based pathway, *VEGFA*, *EPO*, *IL1*, *IL8*, *IL10*, *ADIPOQ* and *CCL2* were involved in receptor binding. *EPO* and *ADIPOQ* are inhibited by TNF (Tumor necrosis factor) , whereas, other genes are activated by TNF. *VEGFA* is activated by SMAD3 (Mothers against decapentaplegic homolog 3) present in the intra-cellular region (Figure 6). Disease pathway analysis showed *IL6*, *EPO*, *ADIPOQ* and *CCR2* to be involved in chronic kidney failure. The hierarchical layout shows that *IL6*, *EPO*, *ADIPOQ* and *CCR2* function by influencing *angiotensin I-converting enzyme* (*ACE*) gene expression. *ACE* further interacts with G-protein beta3-subunit (GNB3) and this interaction is also associated with hypertension in many populations (Figure 7).

### Publication Bias

Thirty six genetic variants were lacking a good number of studies (i.e. less than three studies), thus it was not possible to calculate the publication bias. Of the other nineteen studies, six SNP studies (ADIPOQ rs17300539; RANTES rs2107538; TGF-B1 rs1800468; TGF-B1 rs2241717; VEGFA rs3024997; VEGFA rs3025000) did not account for publication bias as evident from the symmetric funnel plot and thirteen did show evidence of publication bias (Additional file 2).

## Discussion

This meta-analysis, including 55 genetic variants in 18 genes from 34 published studies, explored the association between the genetic variants within the genes involved in inflammatory cytokines and angiogenesis pathways and diabetic nephropathy. The results showed that 11 genetic variants were significantly associated with diabetic nephropathy.

Cytokines act as pleiotropic polypeptides regulating inflammatory and immune responses through actions on cells. The relationships between inflammation and the development and progression of diabetic nephropathy involve complex molecular networks and processes. Many studies have shown a strong association of circulating inflammatory markers and proinflammatory cytokines with the risk of developing of diabetic complications [40]. Evidence from studies where immunosuppressive strategies reduce renal macrophage accumulation and attenuate the development of diabetic nephropathy, support the role of inflammation in diabetic complications [40].

Angiogenesis, the formation of new blood vessels from pre-existing vasculature, has been implicated in the genesis of diverse diabetic complications including diabetic nephropathy. Several studies have reported an increase in potent stimulators of angiogenesis in diabetic nephropathy [41,42]. In addition, the therapeutic efficacies of anti–angiogenic strategies have further demonstrated the involvement of angiogenesis in the progression of diabetic nephropathy [13,43]. In this context, genetics of inflammatory cytokines and angiogenesis is important to analyze the involved genes and their role in susceptibility to diabetic nephropathy.

In this meta-analysis, variants within or near *VEGFA* (two variants), *CCR5* (two variants), *CCL2*, *IL-1*, *MMP9*, *EPO*, *IL-8*, *ADIPOQ* and *IL-10* were significantly associated with diabetic nephropathy. Three genetic variants (two in the *VEGFA* and one in the *EPO* gene) belonged to angiogenesis pathway whereas the other variants were included in inflammatory cytokines. These results support a role of the inflammatory cytokines and angiogenesis pathways in the pathogenesis of diabetic nephropathy.

It has been reported that there is an increased *VEGF* expression in patients with diabetic nephropathy and antibodies against *VEGF* in the early stages of experimental diabetes can ameliorate the renal dysfunction [31]. In this meta-analysis, two genetic variants in *VEGFA* gene were significantly associated with diabetic nephropathy. Both genetic variants were analysed in studies with moderate sample size. and more studies are needed to establish true effect sizes in such cases [44]. T allele of rs833061 genetic variant in *VEGFA* gene was found to increase the risk of diabetic nephropathy whereas Mooyaart et al. [44] in their study considered C allele of rs833061 as having a protective effect.

*CCL2*, also known as *Monocyte chemo-attractant protein-1*(*MCP-1*), is the strongest known chemo-tactic factor for monocytes and is upregulated in diabetic nephropathy [18]. In this meta-analysis, the insertion at *CCL2* rs3917887 showed two fold higher risk of diabetic nephropathy as compared to controls. Chinoy et al. have reported the association *CCL2* rs3917887 with development of inflammatory myopathies [45].

*CCR5* is a β-chemokine receptor involved in a migration of monocytes, NK cells and some T-cells to the inflammation site [20]. *CCR5* rs1799987 was the most studied genetic variant in inflammatory cytokines. This SNP is reported to increase the protein level by increasing the transcriptional activity of the *CCR5* gene [46]. Also, for genetic variant rs1799987 in *CCR5* gene A allele was the risk factor for diabetic nephropathy in our study whereas Mooyaart et al. [44] considered G allele of rs1799987 as a protective allele. The 32 –bp deletion in *CCR5* rs333 changes the open reading frame of the gene resulting in a trunacated protein [47]. This polymorphism showing a significant association with DN in this meta-analysis, is shown to promote renal fibrosis instead of normal tissue repair [48].

*ADIPOQ* encodes adiponectin, a hormone exclusively secreted by the adipose tissue [23]. A allele of rs17300539 in the promoter of *ADIPOQ* is a risk factor for DN. It has been suggested that this genetic variation may contribute to an increased risk of developing nephropathy partly through the increase in adiponectin levels [25].

*IL-8* is a member of the CXC chemokine family and is associated with a variety of proinflammatory activities [49]. rs4073 lies in the regulatory region and increases the protein level. An increase in the urinary excretion of *IL-8* in DN patients has been reported [18]. In their study, Skov et al., showed that *IL-8* is an antibody therapeutic target in inflammatory diseases [49].

The pro-inflammatory cytokine interleukin 1 (IL-1) stimulates kidney mesangial cell proliferation and extracellular matrix expansion, contributing to pathogenesis of DN [30]. In this meta-analysis, there is only one study showing allele IL1B*2 associated with a nearly two fold higher risk of diabetic nephropathy. Additional studies are required to establish the reported risk factor.

*MMP9* rs17576 lies in the substrate binding region and leads to over-accumulation of extracellular matrix, leading to renal damage [18]. Rysz et al. reported an increase in MMP-9 in diabetic nephropathy when compared with diabetes with normal renal function [50].

EPO is a potent angiogenic factor involved in diabetic micro-vascular complications [39]. Garcia et al. reported EPO increased the rate of renal damage [51]. T allele of genetic variant rs1617640 in *EPO* gene was a risk allele for diabetic nephropathy in this meta-analysis. This finding was in agreement with Williams et al. [52] who in their study showed T allele of rs1617640 as a risk factor for diabetic nephropathy. On the contrary, Mooyaart et al. [44] considered T allele to have a protective effect for diabetic nephropathy.

Interleukin-10 (IL-10) fulfils the criteria for an anti-inflammatory and immunosuppressive cytokine [53]. Wong et al. reported a correlation between IL-10 levels and extent of renal damage in diabetic nephropathy [54]. There were two studies investigating the protective effect of T allele of rs1800871 for diabetic nephropathy. Both the studies were done in Tunisian population by the same authors with apparently the same study group (Table 1- ref. 2, 29). Since the methodology and results were identical for the two studies, we considered them as a single study to avoid overestimation of effects.

An important factor that bears on the interpretation of meta-analysis of the genetics of diabetic nephropathy is the adequacy of phenotype definition. This problem is reflected in studies showing that most patients with type 1 diabetes categorized initially as having microalbuminaria, undergo regression to normoalbuminaria [52]. To avoid this problem in our meta-analysis, we only included studies where diabetic nephropathy was defined by macroalbuminaria or ESRD. As with all meta-analysis, one major limitation of this study is publication bias. Only published data in journals was included in this study which may lead to ignoring the negative or non-significant association studies. Heterogeneity between the studies can affect the interpretation of results. One of the potential reason for heterogeneity is the winner’s curse, which appears as an upward bias in the estimated effect of a newly identified allele on disease risk when the study design lacks sufficient statistical power. . The winner’s curse manifests mostly in genome‐wide association (GWA) studies in which 300 000–1 000 000 single‐nucleotide polymorphisms are tested [55]. All the studies included in this meta-analysis were candidate gene based. Further, to minimize the overestimation caused by the winner’s curse, effect sizes and minor allele frequencies were calculated from pooled estimates from the initial reporting study and other subsequent studies. Random effects model was performed to account for any possible heterogeneity.

In this study, enrichment analysis of the genes significantly associated with diabetic nephropathy, generated three functional pathways- GPCR signaling, receptor binding and chronic kidney failure. GPCRs transduce extracellular signals inside the cell through activation of heterotrimeric G proteins and/or via other G protein-independent signaling pathways. Agonist-induced GPCR phosphorylation by G protein-coupled receptor kinases (GRKs) are involved in a number of important systems related to the development and progression of Diabetic nephropathy [56]. Presence of these genes in the functional pathways supports their significant association with diabetic nephropathy as concluded in this meta-analysis. However, further experiments are required to elucidate the exact role of these genes in the pathogenesis of diabetic nephropathy.

Considering a worldwide increase in diabetes, there is an urgent need to provide patients protection from the development and progression of diabetic nephropathy. Identification of genes involved in the primary mechanisms contributing to onset and progression of diabetic nephropathy is important for developing new therapeutic interventions. Inflammatory cytokines and angiogenic factors play a diverse role in the pathogenesis of diabetic nephropathy. The recognition of the genes/genetic variants as having a significant association with risk of diabetic nephropathy will provide new therapeutic targets. Furthermore, this will also help to identify patients with increased risk of diabetic nephropathy.

## Conclusions

This study identified 11 genetic variants in or near 9 genes -*VEGFA*, *CCR5*, *CCL2*, *IL-1*, *MMP9*, *EPO*, *IL-8*, *ADIPOQ* and *IL-10*, significantly associated with diabetic nephropathy. Further studies are required to better understand their functional relevance in the pathogenesis of diabetic nephropathy. These genetic variants within the inflammatory cytokines and angiogensis pathways might be good candidates for identifying genetic predisposition to DN as well as revealing the pathogenesis of diabetic nephropathy.

## Additional files

Additional file 1:
**a:** **Details of SNPs included in the meta-analysis, for genes CCR5, ADIPOQ, IL-8 and CCL2.** **b**: Details of SNPs included in the meta-analysis, for genes IL-10, MMP9 and IL-1. **c**: Details of SNPs included in the meta-analysis, for genes VEGFA, VEGFB and VEGFC. **d**: Details of SNPs included in the meta-analysis, for genes IL-6, CCR2, EPO, TNFα and CCL5. **e**: Details of SNPs included in the meta-analysis, for genes TGF-B1, TGF-BR1 and TGF-BR2. Additional file 2:
**Funnel plots of nineteen SNPs with more than two studies.** **a**: Funnel plot of ADIPOQ rs17300539. **b**: Funnel plot of CCR5 rs333. **c**: Funnel plot of CCR5 rs2734648. **d**: Funnel plot of EPO rs1617640. **e**: Funnel plot of CCR5 rs1799987. **f**: Funnel plot of RANTES rs2107538. **g**: Funnel plot of TGF-B1 rs1800468. **h**: Funnel plot of TGF-B1 rs1800469. **i**: Funnel plot of TGF-B1 rs2241717. **j**: Funnel plot of TGF-B1 rs8179181. **k**: Funnel plot of TGF-BR1 rs928180. **l**: Funnel plot of TGF-BR1 rs1571589. **m**: Funnel plot of TGF-B1 rs1800471. **n**: Funnel plot of TGF-B1 rs1800470. **o**: Funnel plot of VEGFA rs3024997. **p**: Funnel plot of VEGFA rs3025000. **q**: Funnel plot of VEGFA rs2146323. **r**: Funnel plot of VEGFB rs12366035. **s**: Funnel plot of VEGFC rs585706.

## Abbreviation

DM: Diabetes mellitus
T1DM: Type 1 Diabetes mellitus
T2DM: Type 2 diabetes mellitus
DN: Diabetic Nephropathy
SNP: Single nucleotide Polymorphism
DNA: Deoxyribonucleic acid
bp: Base pair
OR: Odds ratio
CI: Confidence interval
HWE: Hardy–Weinberg equilibrium
ESRD: End stage renal disease
NK Cells: Natural killer cells
STZ: Streptozotocin
VEGFA: Vascular endothelial growth factor A
CCR5: Chemokine (C-C motif) receptor type 5
CCL2: Chemokine ligand 2
IL1: Interleukin-1
MMP9: Matrix metallopeptidase 9
EPO: Erythropoietin
IL8: Interleukin 8
ADIPOQ: Adiponectin C1Q and collagen domain containing
IL10: Interleukin 10
GRKs: G protein-coupled receptor kinases
GPCRs: G protein-coupled receptors
IL1B: Interleukin-1 beta
IL6: Interleukin 6
MCP1: Monocyte chemoattractant protein-1
BMP4: Bone morphogenetic protein 4
BMP2: Bone morphogenetic protein 2
BMP7: Bone morphogenetic protein 7
TGFB1: Transforming growth factor beta 1
JAG1: jagged 1 protein
SMAD2: Mothers against decapentaplegic homolog 2
SMAD3: Mothers against decapentaplegic homolog 3
ENPP1: Ectonucleotide pyrophosphatase/phosphodiesterase family member 1
TCF7L2: transcription factor 7-like 2
ACE: angiotensin I converting enzyme
APOE: Apolipoprotein E
SERPINEI: Serpin peptidase Inhibitor, Clade E (Nexin, Plasminogen activator Inhibitor Type 1), Member 1
ICAM1: Intercellular Adhesion Molecule 1
TNF: Tumor necrosis factor
ADAM10: A Disintegrin and metalloproteinase domain-containing protein 10
VDR: Vitamin D (1,25- Dihydroxyvitamin D3) Receptor
NPY: Neuropeptide Y
GNB3: Guanine nucleotide-binding protein G(I)/G(S)/G(T) subunit beta-3
REN: Rennin
CCL5: Chemokine ligand 5
CCR2: Chemokine (C-C motif) receptor 2
AGT: Angiotensinogen
AGTR1: Angiotensin II receptor
CTGF: Connective tissue growth factor
MTHFR: Methylenetetrahydrofolate reductase
NOS3: Nitric oxide synthase 3
SLC12A3: Solute carrier family 12 (Sodium/Chloride Transporter), member 3

## References

[1] International Diabetes Federation: *IDF Diabetes Atlas.* 6th edition. Brussels, Belgium: International Diabetes Federation; 2013. [http://www.idf.org/diabetesatlas]

[2] Ezzidi I, Mtiraoui N, Kacem M, Mallat SG, Mohamed MB, Chaieb M, Mahjoub T, Almawi WY (2009). Interleukin-10–592C/A, −819C/T and –1082A/G promoter variants affect the susceptibility to nephropathy in Tunisian type 2 diabetes (T2DM) patients. Clin Endocrinol.

[3] Zhang D, Efendic S, Brismar K, Gu HF (2010). Effects of MCF2L2, ADIPOQ and SOX2 genetic polymorphisms on the development of nephropathy in type 1 Diabetes Mellitus. BMC Med Genet.

[4] Freedman BI, Bostrom M, Daeihagh P, Bowden DW (2007). Genetic Factors in Diabetic Nephropathy. Clin J Am Soc Nephrol.

[5] Blech I, Katzenellenbogen M, Katzenellenbogen A, Wainstein J, Rubinstein A, Harman-Boehm I, Cohen J, Pollin TI, Glaser B (2011). Predicting diabetic nephropathy using a multifactorial genetic model. PLoS One.

[6] Xu ZG, Lanting L, Vaziri ND, Li Z, Sepassi L, Rodriguez-Iturbe B, Natarajan R (2005). Upregulation of angiotensin II type I receptor, inflammatory mediators, and enzymes of arachidonate metabolism in obese Zucker rat kidney: reversal by angiotensin II type 1 receptor blockade. Circulation.

[7] Tashiro K, Koyanagi I, Saitoh A, Shimizu A, Shike T, Ishiguro C, Koizumi M, Funabiki K, Horikoshi S, Shirato I, Tomino Y (2002). Urinary levels of monocyte chemoattractant protein-1 (MCP-1) and inter-leukin-8 (IL-8), and renal injuries in patients with type 2 diabetic nephropathy. J Clin Lab Anal.

[8] Lewis A, Steadman R, Manley P, Craig K, de la Motte C, Hascall V, Phillips AO (2008). Diabetic nephropathy, inflammation, hyaluronan and interstitial fibrosis, *Histol*. Histopathol.

[9] Nyengaard JR, Rasch R (1993). The impact of experimental diabetes mellitus in rats on glomerular capillary number and sizes. Diabetologia.

[10] Sharma K, Ziyadeh FN (1995). Hyperglycemia and diabetic kidney disease. The case for transforming growth factor-beta as a key mediator. Diabetes.

[11] Kim NH, Kim KB, Kim DL, Kim SG, Choi KM, Baik SH, Choi DS, Kang YS, Han SY, Han KH, Ji YH, Cha DR (2004). Plasma and urinary vascular endothelial growth factor and diabetic nephropathy in type 2 diabetes mellitus. Diabet Med.

[12] Chen S, Ziyadeh FN (2008). Vascular endothelial growth factor and diabetic nephropathy. Curr Diab Rep.

[13] Celec P, Hodosy J, Gardlík R, Behuliak M, Pálffy R, Pribula M, Jáni P, Turňa J, Sebeková K (2012). The effects of anti-inflammatory and anti- angiogenic DNA vaccination on diabetic nephropathy in rats. Hum Gene Ther.

[14] Higgins JP, Thompson SG, Deeks JJ, Altman DG (2003). Measuring inconsistency in meta-analyses. Brit Med J.

[15] Kavvoura FK, Ioannidis JPA (2008). Methods for meta-analysis in genetic association studies: a review of their potential and pitfalls. Hum Genet.

[16] Pettigrew KA, McKnight AJ, Patterson CC, Kilner J, Sadlier DM, Maxwell AP (2010). Resequencing of the CCL5 and CCR5 genes and investigation of variants for association with diabetic nephropathy. J Hum Genet.

[17] Tregouet DA, Groop PH, McGinn S, Forsblom C, Hadjadj S, Marre M, Parving HH, Tarnow L, Telgmann R, Godefroy T, Nicaud V, Rousseau R, Parkkonen M, Hoverfalt A, Gut I, Heath S, Matsuda F, Cox R, Kazeem G, Farrall M, Gauguier D, Brand-Herrmann SM, Cambien F, Lathrop M, Vionnet N (2008). G/T substitution in intron 1 of the UNC13B gene is associated with increased risk of nephropathy in patients with type 1 diabetes. Diabetes.

[18] Ahluwalia TS, Khullar M, Ahuja M, Kohli HS, Bhansali A, Mohan V, Venkatesan R, Rai TS, Sud K, Singal PK (2009). Common Variants of Inflammatory Cytokine Genes Are Associated with Risk of Nephropathy in Type 2 Diabetes among Asian Indians. PLoS One.

[19] Mlynarski WM, Placha GP, Wolkow PP, Bochenski JP, Warram JH, Krolewski AS (2005). Risk of diabetic nephropathy in type 1 diabetes is associated with functional polymorphisms in RANTES receptor gene (CCR5): a sex-specific effect. Diabetes.

[20] Buraczynska M, Zukowski P, Wacinski P, Berger-Smyka B, Dragan M, Mozul S (2012). Chemotactic cytokine receptor 5 gene polymorphism: Relevance to microvascular complications in type 2 diabetes. Cytokine.

[21] Nakajima K, Tanaka Y, Nomiyama T, Ogihara T, Ikeda F, Kanno R, Iwashita N, Sakai K, Watada H, Onuma T, Kawamori R (2003). RANTES promoter genotype is associated with diabetic nephropathy in type 2 diabetic subjects. Diabetes Care.

[22] Prasad P, Tiwari AK, Kumar KMP, Ammini AC, Gupta A, Gupta R, Thelma BK (2007). Association of TGFbeta1, TNFalpha, CCR2 and CCR5 gene polymorphisms in type-2 diabetes and renal insufficiency among Asian Indians. BMC Med Genet.

[23] Zhang D, Ma J, Brismar K, Efendic S, Gu HF (2009). A single nucleotide polymorphism alters the sequence of SP1 binding site in the adiponectin promoter region and is associated with diabetic nephropathy among type 1 diabetic patients in the Genetics of Kidneys in Diabetes Study. J Diabetes Complicat.

[24] Wu LSH, Hsieh CH, Pei D, Hung YJ, Kuo SW, Lin E (2009). Association and interaction analyses of genetic variants in ADIPOQ, ENPP1, GHSR, PPARγ and TCF7L2 genes for diabetic nephropathy in a Taiwanese population with type 2 diabetes. Nephrol Dial Transplant.

[25] Vionnet N, Tregouët D, Kazeem G, Gut I, Groop PH, Tarnow L, Parving HH, Hadjadj S, Forsblom C, Farrall M, Gauguier D, Cox R, Matsuda F, Heath S, Thevard A, Rousseau R, Cambien F, Marre M, Lathrop M (2006). Analysis of 14 Candidate Genes for Diabetic Nephropathy on Chromosome 3q in European Populations Strongest Evidence for Association With a Variant in the Promoter Region of the Adiponectin Gene. Diabetes.

[26] Prior SL, Javid J, Gill GV, Bain SC, Stephens JW (2008). The adiponectin rs17300539 G > A variant and nephropathy risk. Kidney Int.

[27] Joo KW, Hwang YH, Kim JH, Oh KH, Kim H, Shin HD, Chung WK, Yang J, Park KS, Ahn C (2007). MCP-1 and RANTES Polymorphisms in Korean Diabetic End-Stage Renal Disease. J Korean Med Sci.

[28] Arababadi MK, Mirzaei MR, Sajadi SMA, Hassanshahi G, Ahmadabadi BN, Salehabadi VA, Derakhshan R, Kennedy D (2012). Interleukin (IL)-10 gene polymorphisms are associated with type 2 diabetes with and without nephropathy: A study of patients from the southest region of Iran. Inflammation.

[29] Mtiraoui N, Ezzidi I, Kacem M, Ben Hadj Mohamed M, Chaieb M, Haj Jilani AB, Mahjoub T, Almawi WY (2009). Predictive value of interleukin-10 promoter genotypes and haplotypes in determining the susceptibility to nephropathy in type 2 diabetes patients. Diabetes Metab Res Rev.

[30] Loughrey BV, Maxwell AP, Fogarty DG, Middleton D, Harron JC, Patterson CC, Darke C, Savage DA (1998). An interleukin 1B allele, which correlates with a high Secretor phenotype, is associated with diabetic Nephropathy. Cytokine.

[31] Yang B, Cross DF, Ollerenshaw M, Millward BA, Demaine AG (2003). Polymorphisms of the vascular endothelial growth factor and susceptibility to diabetic microvascular complications in patients with type 1 diabetes mellitus. J Diabetes Complicat.

[32] Buraczynska M, Ksiazek P, Gaszczyk IB, Jozwiak L (2007). Association of the VEGF gene polymorphism with diabetic retinopathy in type 2 diabetes patients. Nephrol Dial Transplant.

[33] McKnight A-J, Maxwell AP, Patterson CC, Brady HR, Savage DA (2007). Association of VEGF-1499C[right arrow]T polymorphism with diabetic nephropathy in type 1 diabetes mellitus. J Diabetes Complicat.

[34] Kim HW, Ko GJ, Kang YS, Lee MH, Song HK, Kim HK, Cha DR (2009). Role of the VEGF 936 C/T polymorphism in diabetic microvascular complications in type 2 diabetic patients. Nephrology.

[35] Ng DP, Nurbaya S, Ye S, Krolewski AS (2008). An IL-6 haplotype on human chromosome 7p21 confers risk for impaired renal function in type 2 diabetes. Kidney Int.

[36] Jahromi MM, Millward BA, Demaine AG (2010). Significant correlation between association of polymorphism in codon 10 of transforming growth factor-β1 T(29) C with type 1 diabetes and patients with nephropathy disorder. J Interf Cytokine Res.

[37] Ng DPK, Warram JH, Krolewski AS (2003). TGF- β1 as a genetic susceptibility locus for advanced diabetic nephropathy in type 1 diabetes mellitus: An investigation of multiple known DNA sequence variants. Am J Kidney Disease.

[38] Salgado-V A, Martinez JA, Rosas M, Garcia-Mena J, Utrera-Barillas D, Gomez-Diaz R, Pena JE, Parra EJ, Cruz M (2010). Association of polymorphisms within the transforming growth factor-β1 gene with diabetic nephropathy and serum cholesterol and triglyceride concentrations. Nephrology.

[39] Tong Z, Yang Z, Patel S, Chen H, Gibbs D, Yang X, Hau VS, Kaminoh Y, Harmon J, Pearson E, Buehler J, Chen Y, Yu B, Tinkham NH, Zabriskie NA, Zeng J, Luo L, Sun JK, Prakash M, Hamam RN, Tonna S, Constantine R, Ronquillo CC, Sadda S, Avery RL, Brand JM, London N, Anduze AL, King GL, Bernstein PS (2008). Promoter polymorphism of the erythropoietin gene in severe diabetic eye and kidney complications. Proc Natl Acad Sci U S A.

[40] Elmarakby AA, Sullivan JC (2012). Relationship between oxidative stress and inflammatory cytokines in diabetic nephropathy. Cardiovasc Ther.

[41] Nasu T, Maeshima Y, Kinomura M, Hirokoshi-Kawahara K, Tanabe K, Sugiyama H, Sonoda H, Sato Y, Makino H (2009). Vasohibin-1, a negative feedback regulator of angiogenesis, ameliorates renal alterations in a mouse model of diabetic nephropathy. Diabetes.

[42] Sonezaki K, Maezawa Y, Takemoto M, Kobayashi K, Tokuyama T, Takada-Watanabe A, Simoyama T, Sato S, Saito Y, Yokote K (2013). Alteration of VEGF and Angiopoietins Expressions in Diabetic Glomeruli Implicated in the Development of Diabetic Nephropathy. Advanced Studies Med Sci.

[43] Zent R, Pozzi A (2007). Angiogenesis in diabetic nephropathy. Semin Nephrol.

[44] Mooyaart AL, Valk EJ, van Es LA, Bruijn JA, de Heer E, Freedman BI, Dekkers OM, Baelde HJ (2011). Genetic associations in diabetic nephropathy: a meta-analysis. Diabetologia.

[45] Chinoy H, Salway F, Fertig N, Tait BD, Oddis CV, Ollier WE, Cooper RG (2007). Monocyte chemotactic protein-1 single nucleotide polymorphisms do not confer susceptibility for the development of adult onset polymyositis/dermatomyositis in UK Caucacians. Rheumatology.

[46] Narvatilova Z (2006). Polymorphisms in CCL2 & CCL5 chemikines/chemokine receptors genes and their association with diseases. Biomed Pap Med Fac Univ Palacky Oloumuc Czech Repub.

[47] Gonzalez E, Dhanda R, Bamshad M, Mummidi S, Geevarghese R, Catano G, Anderson SA, Walter EA, Stephan KT, Hammer MF, Mangano A, Sen L, Clark RA, Ahuja SS, Dolan MJ, Ahuja SK (2001). Global survey of genetic variation in CCR5, RANTES and MIP-alpha:impact on the epidemiology of the HIV-1 pandemic. Proc Natl Acad Sci U S A.

[48] Vielhauer V, Anders HJ, Mack M, Cihak J, Strutz F, Stangassinger M, Luckow B, Gröne HJ, Schlöndorff D (2001). Obstructive nephropathy in the mouse: progressive fibrosis correlates with tubulointerstitial chemokine expression and accumulation of CC chemokine receptor 2- and 5- positive leukocytes. J Am Soc Nephrol.

[49] Skov L, Beurskens FJ, Zachariae CO, Reitamo S, Teeling J, Satijn D, Knudsen KM, Boot EP, Hudson D, Baadsgaard O, Parren PW, van de Winkel JG (2008). IL-8 as Antibody Therapeutic Target in Inflammatory Diseases: Reduction of Clinical Activity in Palmoplantar Pustulosis. J Immunol.

[50] Rysz J, Banach M, Stolarek RA, Pasnik J, Cialkowska-Rysz A, Koktysz R, Piechota M, Baj Z (2007). Serum matrix metalloproteinases MMP-2 and MMP-9 and metalloproteinase tissue inhibitors TIMP-1 and TIMP-2 in diabetic nephropathy. J Nephrol.

[51] Garcia DL, Anderson S, Rennke HG, Brenner BM (1988). Anemia lessens and its prevention with recombinant human erythropoietin worsens glomerular injury and hypertension in rats with reduced renal mass. Proc Natl Acad Sci U S A.

[52] Williams WW, Salem RM, McKnight AJ, Sandholm N, Forsblom C, Taylor A, Guiducci C, McAteer JB, McKay GJ, Isakova T, Brennan EP, Sadlier DM, Palmer C, Soderlund J, Fagerholm E, Harjutsalo V, Lithovius R, Gordin D, Hietala K, Kyto J, Parkkonen M, Rosengard-Barlund M, Thorn L, Syreeni A, Tolonen N, Saraheimo M, Waden J, Pitkaniemi J, Sarti C, Tuomilehto J, Tryggvason K (2012). Association testing of previously reported variants in a large case–control meta-analysis of diabetic nephropathy. Diabetes.

[53] Myśliwska J, Zorena K, Semetkowska-Jurkiewicz E, Rachoń D, Suchanek H, Myśliwski A (2005). High levels of circulating interleukin-10 in diabetic nephropathy patients. Eur Cytokine Netw.

[54] Wong CK, Ho AW, Tong PC, Yeung CY, Kong AP, Lun SW, Chan JC, Lam CW (2007). Aberrant activation profile of cytokines and mitogen-activated protein kinases in type 2 diabetic patients with nephropathy. Clin Exp Immunol.

[55] Nakaoka H, Inoue I (2010). The Winner’s Curse. eLS.

[56] Wang FL, Tang LQ, Wei W (2012). The connection between GRKs and various signaling pathways involved in diabetic nephropathy. Mol Biol Rep.

