# Supplementary figures and images for: Meta-analysis of diabetic nephropathy associated genetic variants in inflammation and angiogenesis involved in different biochemical pathways
Source: BMC Med Genet. 2014 Oct 4;15:103. doi: 10.1186/s12881-014-0103-8 (PMC4411872; doi:10.1186/s12881-014-0103-8)

**Funnel Plot of Standard Error by Log odds ratio**

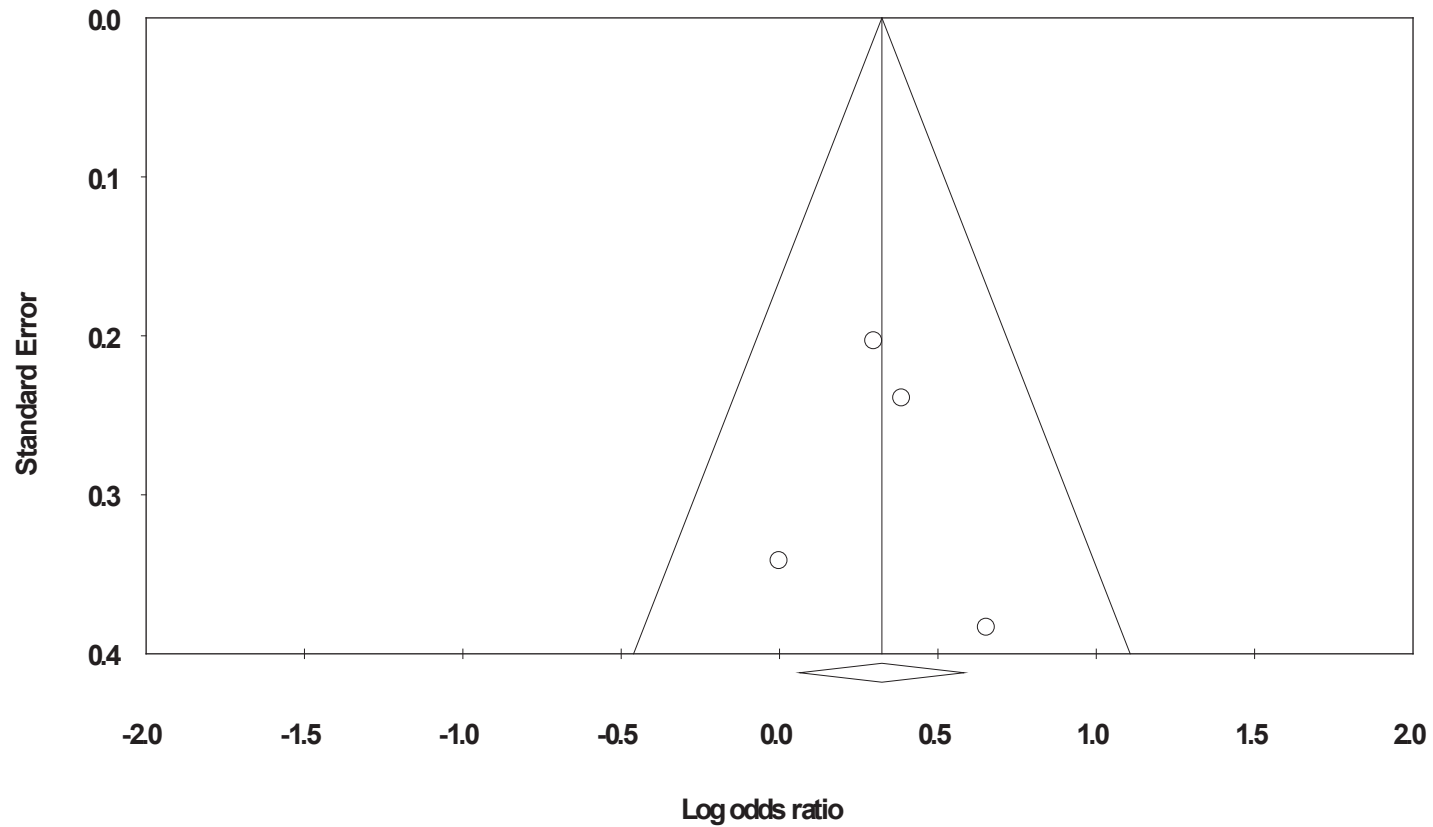

Supplement: Additional file 2: — Funnel plots of nineteen SNPs with more than two studies. a: Funnel plot of ADIPOQ rs17300539. b: Funnel plot of CCR5 rs333. c: Funnel plot of CCR5 rs2734648. d: Funnel plot of EPO rs1617640. e: Funnel plot of CCR5 rs1799987. f: Funnel plot of RANTES rs2107538. g: Funnel plot of TGF-B1 rs1800468. h: Funnel plot of TGF-B1 rs1800469. i: Funnel plot of TGF-B1 rs2241717. j: Funnel plot of TGF-B1 rs8179181. k: Funnel plot of TGF-BR1 rs928180. l: Funnel plot of TGF-BR1 rs1571589. m: Funnel plot of TGF-B1 rs1800471. n: Funnel plot of TGF-B1 rs1800470. o: Funnel plot of VEGFA rs3024997. p: Funnel plot of VEGFA rs3025000. q: Funnel plot of VEGFA rs2146323. r: Funnel plot of VEGFB rs12366035. s: Funnel plot of VEGFC rs585706. [file 12881_2014_103_MOESM2_ESM.zip › Additional file 2/7053225111232618_add2a.pdf]

**Funnel Plot of Standard Error by Log odds ratio**

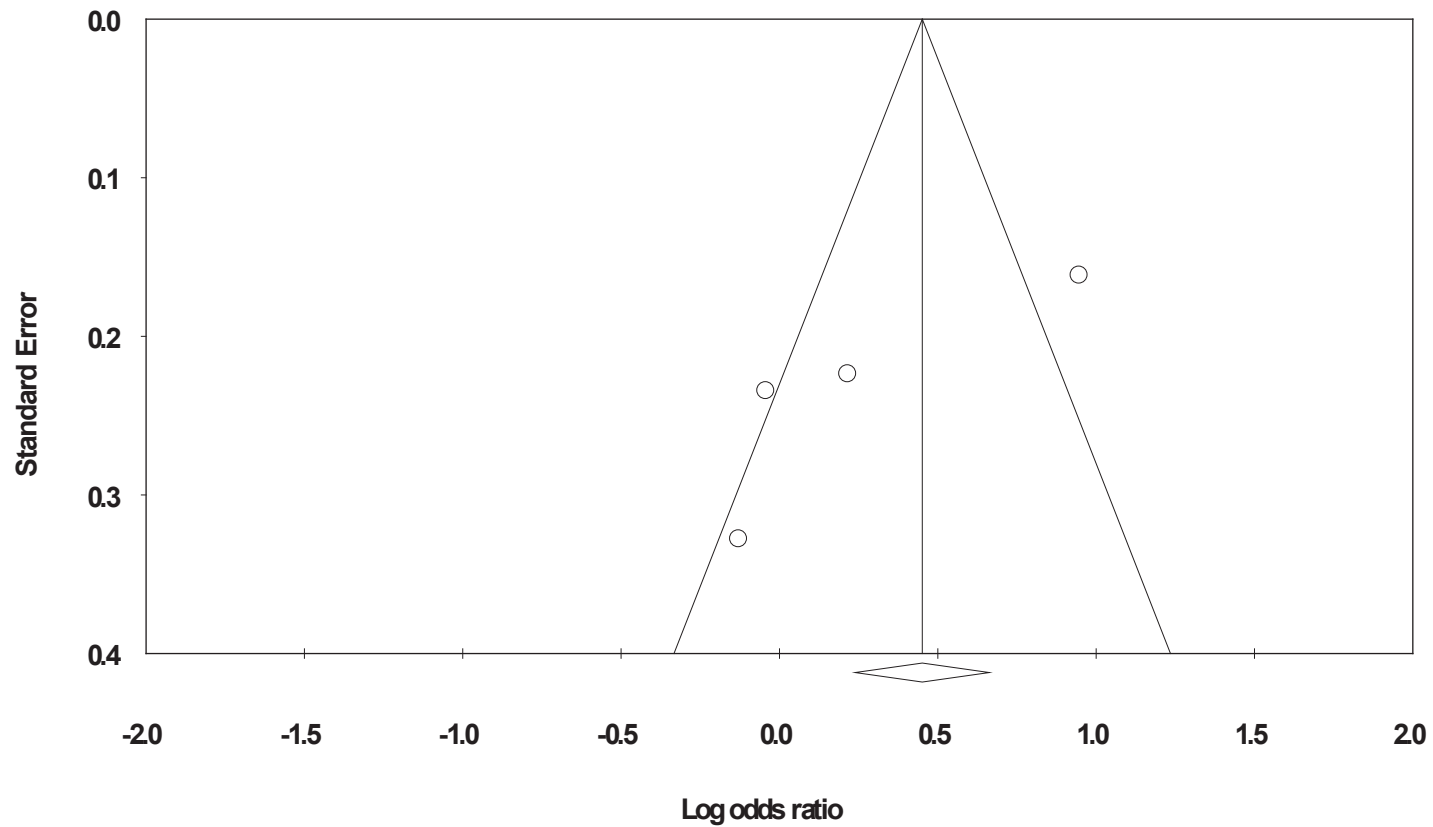

Supplement: Additional file 2: — Funnel plots of nineteen SNPs with more than two studies. a: Funnel plot of ADIPOQ rs17300539. b: Funnel plot of CCR5 rs333. c: Funnel plot of CCR5 rs2734648. d: Funnel plot of EPO rs1617640. e: Funnel plot of CCR5 rs1799987. f: Funnel plot of RANTES rs2107538. g: Funnel plot of TGF-B1 rs1800468. h: Funnel plot of TGF-B1 rs1800469. i: Funnel plot of TGF-B1 rs2241717. j: Funnel plot of TGF-B1 rs8179181. k: Funnel plot of TGF-BR1 rs928180. l: Funnel plot of TGF-BR1 rs1571589. m: Funnel plot of TGF-B1 rs1800471. n: Funnel plot of TGF-B1 rs1800470. o: Funnel plot of VEGFA rs3024997. p: Funnel plot of VEGFA rs3025000. q: Funnel plot of VEGFA rs2146323. r: Funnel plot of VEGFB rs12366035. s: Funnel plot of VEGFC rs585706. [file 12881_2014_103_MOESM2_ESM.zip › Additional file 2/7053225111232618_add2b.pdf]

**Funnel Plot of Standard Error by Log odds ratio**

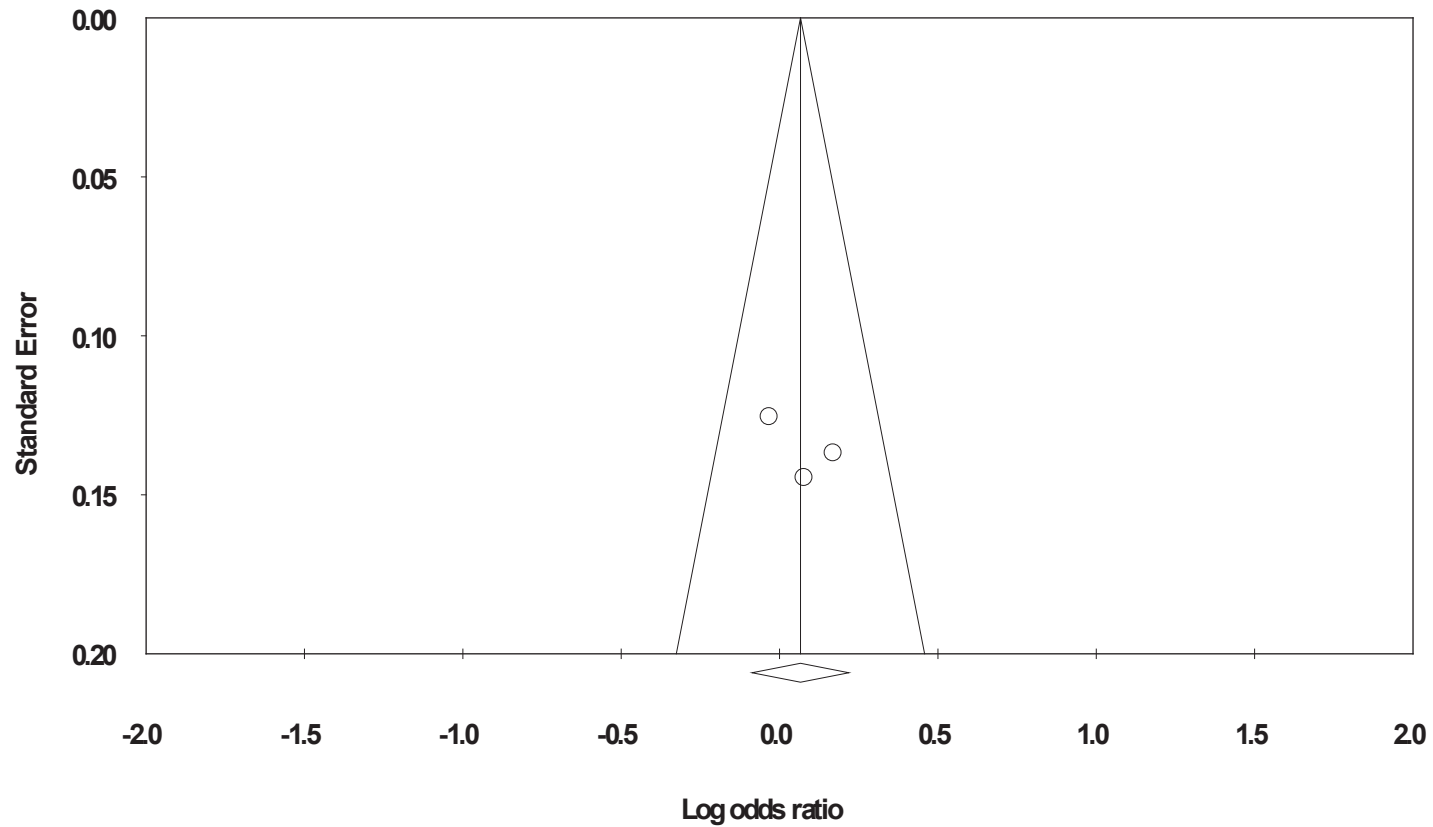

Supplement: Additional file 2: — Funnel plots of nineteen SNPs with more than two studies. a: Funnel plot of ADIPOQ rs17300539. b: Funnel plot of CCR5 rs333. c: Funnel plot of CCR5 rs2734648. d: Funnel plot of EPO rs1617640. e: Funnel plot of CCR5 rs1799987. f: Funnel plot of RANTES rs2107538. g: Funnel plot of TGF-B1 rs1800468. h: Funnel plot of TGF-B1 rs1800469. i: Funnel plot of TGF-B1 rs2241717. j: Funnel plot of TGF-B1 rs8179181. k: Funnel plot of TGF-BR1 rs928180. l: Funnel plot of TGF-BR1 rs1571589. m: Funnel plot of TGF-B1 rs1800471. n: Funnel plot of TGF-B1 rs1800470. o: Funnel plot of VEGFA rs3024997. p: Funnel plot of VEGFA rs3025000. q: Funnel plot of VEGFA rs2146323. r: Funnel plot of VEGFB rs12366035. s: Funnel plot of VEGFC rs585706. [file 12881_2014_103_MOESM2_ESM.zip › Additional file 2/7053225111232618_add2c.pdf]

**Funnel Plot of Standard Error by Log odds ratio**

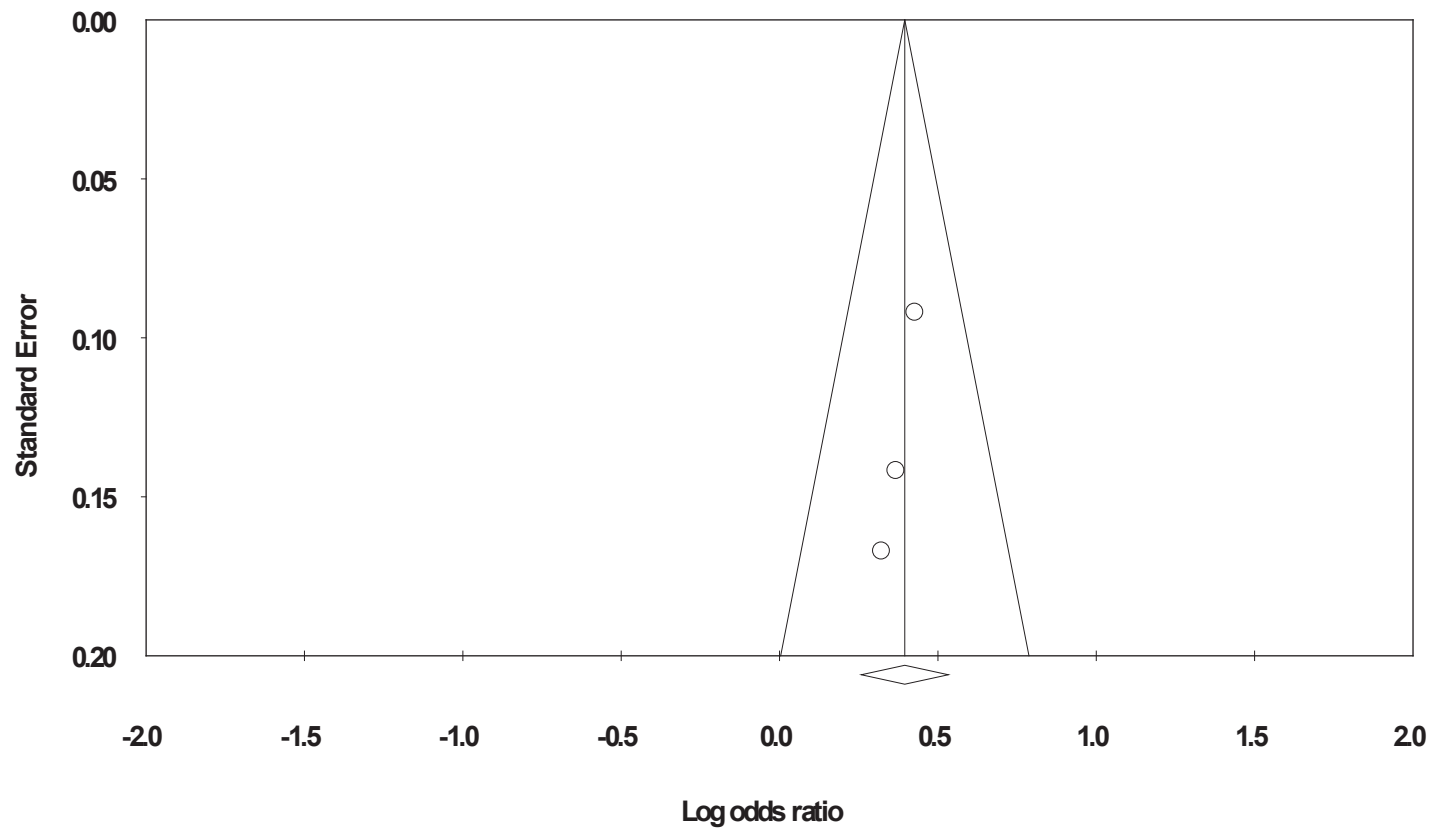

Supplement: Additional file 2: — Funnel plots of nineteen SNPs with more than two studies. a: Funnel plot of ADIPOQ rs17300539. b: Funnel plot of CCR5 rs333. c: Funnel plot of CCR5 rs2734648. d: Funnel plot of EPO rs1617640. e: Funnel plot of CCR5 rs1799987. f: Funnel plot of RANTES rs2107538. g: Funnel plot of TGF-B1 rs1800468. h: Funnel plot of TGF-B1 rs1800469. i: Funnel plot of TGF-B1 rs2241717. j: Funnel plot of TGF-B1 rs8179181. k: Funnel plot of TGF-BR1 rs928180. l: Funnel plot of TGF-BR1 rs1571589. m: Funnel plot of TGF-B1 rs1800471. n: Funnel plot of TGF-B1 rs1800470. o: Funnel plot of VEGFA rs3024997. p: Funnel plot of VEGFA rs3025000. q: Funnel plot of VEGFA rs2146323. r: Funnel plot of VEGFB rs12366035. s: Funnel plot of VEGFC rs585706. [file 12881_2014_103_MOESM2_ESM.zip › Additional file 2/7053225111232618_add2d.pdf]

**Funnel Plot of Standard Error by Log odds ratio**

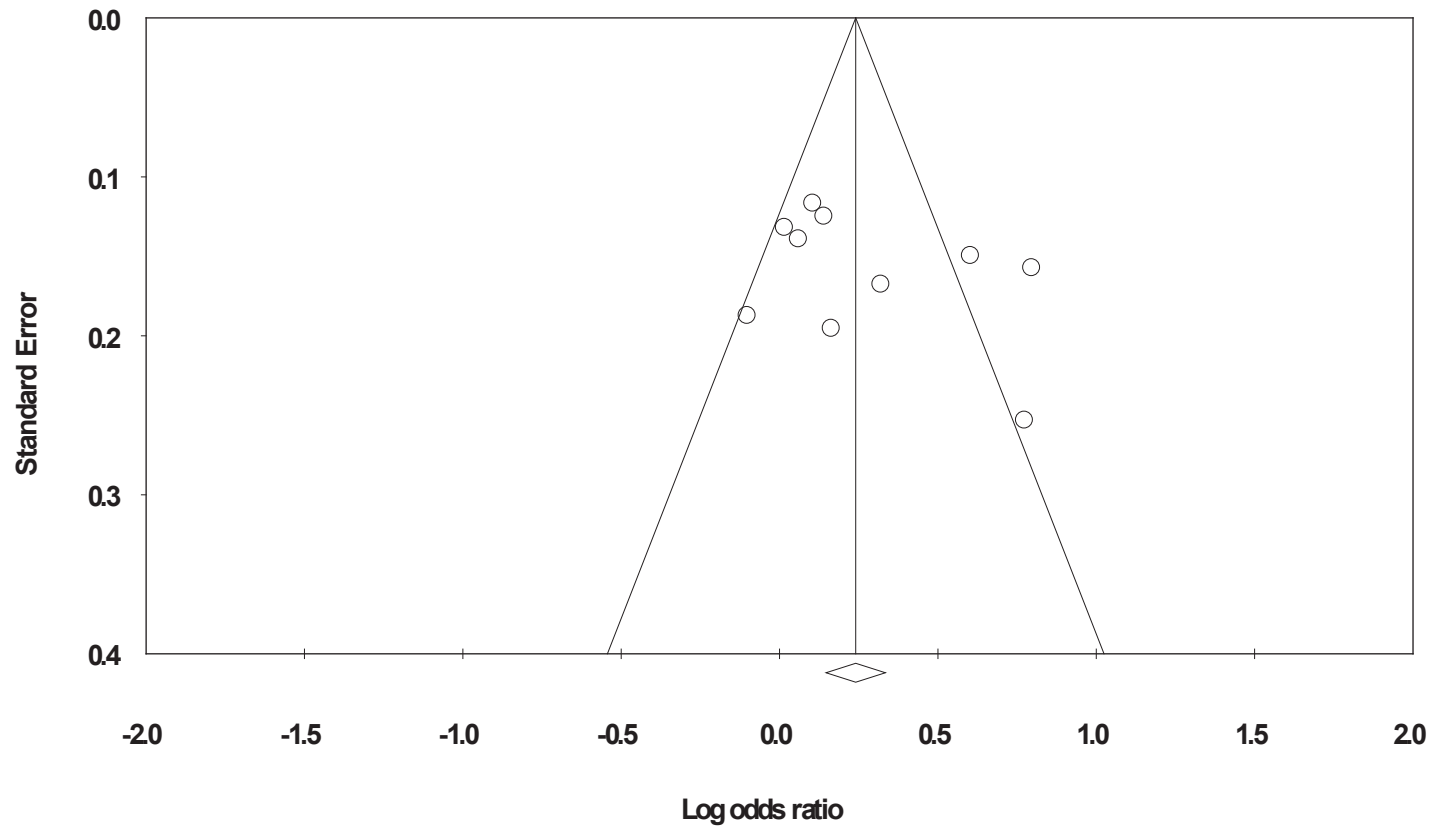

Supplement: Additional file 2: — Funnel plots of nineteen SNPs with more than two studies. a: Funnel plot of ADIPOQ rs17300539. b: Funnel plot of CCR5 rs333. c: Funnel plot of CCR5 rs2734648. d: Funnel plot of EPO rs1617640. e: Funnel plot of CCR5 rs1799987. f: Funnel plot of RANTES rs2107538. g: Funnel plot of TGF-B1 rs1800468. h: Funnel plot of TGF-B1 rs1800469. i: Funnel plot of TGF-B1 rs2241717. j: Funnel plot of TGF-B1 rs8179181. k: Funnel plot of TGF-BR1 rs928180. l: Funnel plot of TGF-BR1 rs1571589. m: Funnel plot of TGF-B1 rs1800471. n: Funnel plot of TGF-B1 rs1800470. o: Funnel plot of VEGFA rs3024997. p: Funnel plot of VEGFA rs3025000. q: Funnel plot of VEGFA rs2146323. r: Funnel plot of VEGFB rs12366035. s: Funnel plot of VEGFC rs585706. [file 12881_2014_103_MOESM2_ESM.zip › Additional file 2/7053225111232618_add2e.pdf]

**Funnel Plot of Standard Error by Log odds ratio**

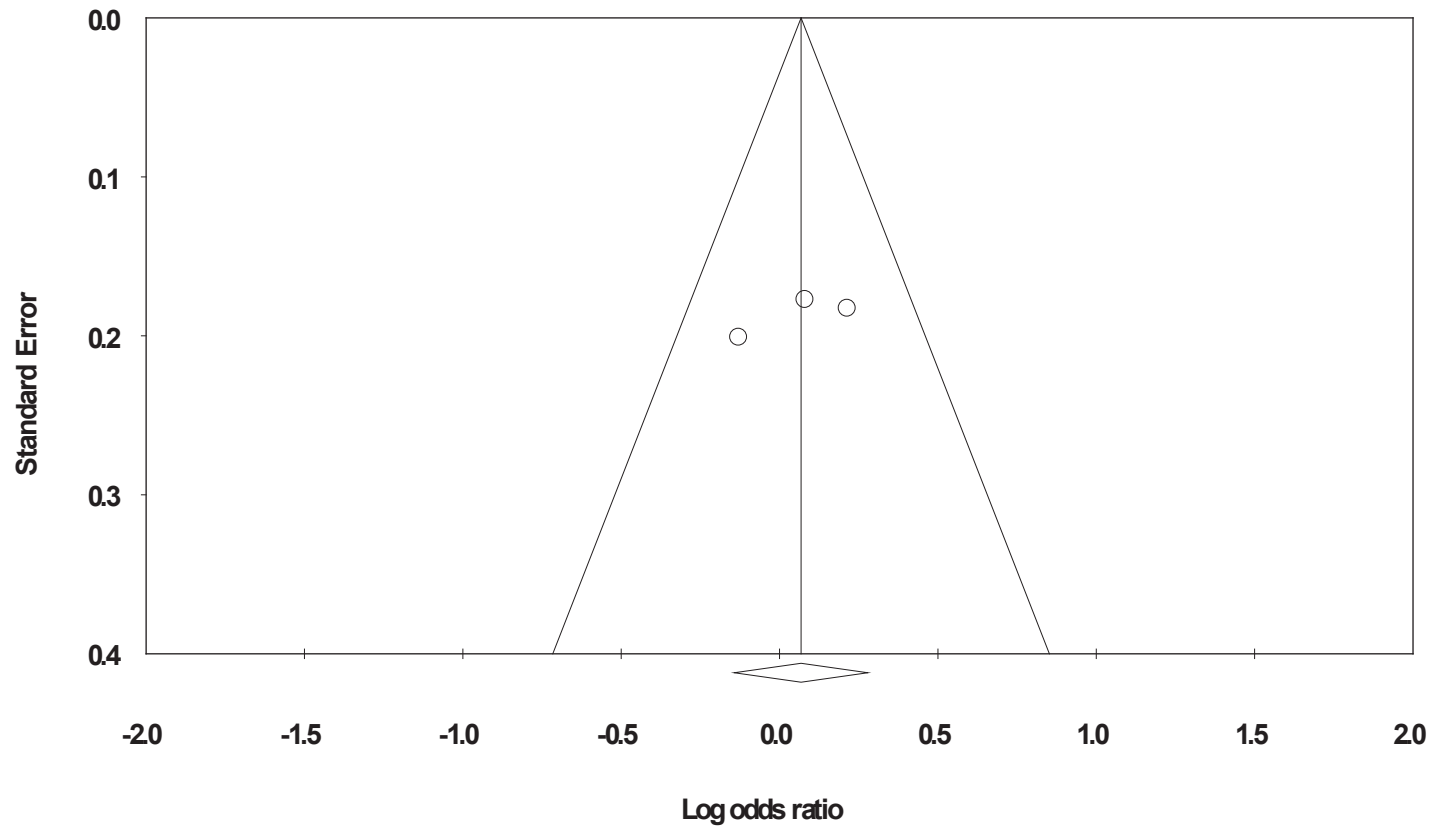

Supplement: Additional file 2: — Funnel plots of nineteen SNPs with more than two studies. a: Funnel plot of ADIPOQ rs17300539. b: Funnel plot of CCR5 rs333. c: Funnel plot of CCR5 rs2734648. d: Funnel plot of EPO rs1617640. e: Funnel plot of CCR5 rs1799987. f: Funnel plot of RANTES rs2107538. g: Funnel plot of TGF-B1 rs1800468. h: Funnel plot of TGF-B1 rs1800469. i: Funnel plot of TGF-B1 rs2241717. j: Funnel plot of TGF-B1 rs8179181. k: Funnel plot of TGF-BR1 rs928180. l: Funnel plot of TGF-BR1 rs1571589. m: Funnel plot of TGF-B1 rs1800471. n: Funnel plot of TGF-B1 rs1800470. o: Funnel plot of VEGFA rs3024997. p: Funnel plot of VEGFA rs3025000. q: Funnel plot of VEGFA rs2146323. r: Funnel plot of VEGFB rs12366035. s: Funnel plot of VEGFC rs585706. [file 12881_2014_103_MOESM2_ESM.zip › Additional file 2/7053225111232618_add2f.pdf]

**Funnel Plot of Standard Error by Log odds ratio**

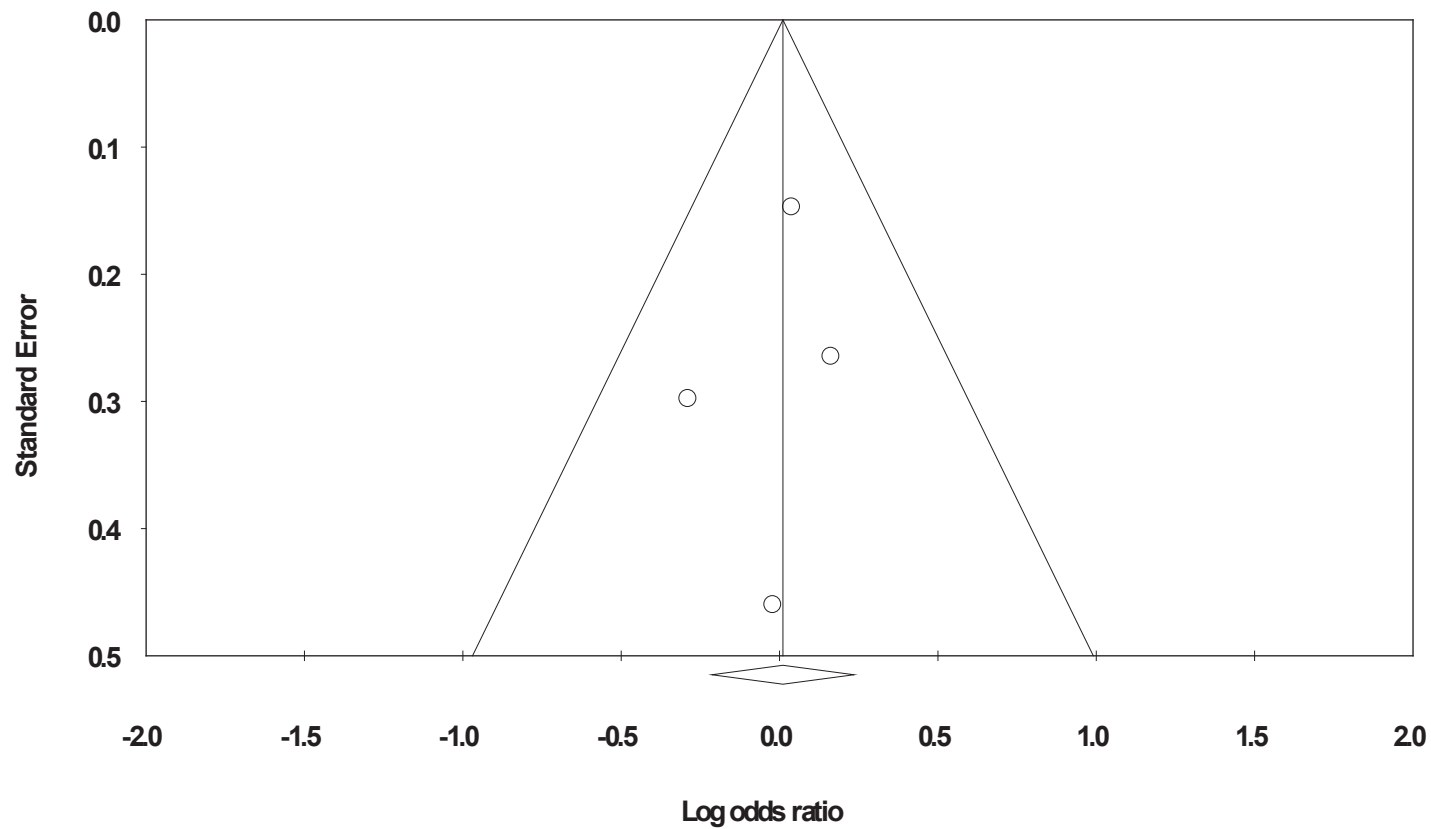

Supplement: Additional file 2: — Funnel plots of nineteen SNPs with more than two studies. a: Funnel plot of ADIPOQ rs17300539. b: Funnel plot of CCR5 rs333. c: Funnel plot of CCR5 rs2734648. d: Funnel plot of EPO rs1617640. e: Funnel plot of CCR5 rs1799987. f: Funnel plot of RANTES rs2107538. g: Funnel plot of TGF-B1 rs1800468. h: Funnel plot of TGF-B1 rs1800469. i: Funnel plot of TGF-B1 rs2241717. j: Funnel plot of TGF-B1 rs8179181. k: Funnel plot of TGF-BR1 rs928180. l: Funnel plot of TGF-BR1 rs1571589. m: Funnel plot of TGF-B1 rs1800471. n: Funnel plot of TGF-B1 rs1800470. o: Funnel plot of VEGFA rs3024997. p: Funnel plot of VEGFA rs3025000. q: Funnel plot of VEGFA rs2146323. r: Funnel plot of VEGFB rs12366035. s: Funnel plot of VEGFC rs585706. [file 12881_2014_103_MOESM2_ESM.zip › Additional file 2/7053225111232618_add2g.pdf]

**Funnel Plot of Standard Error by Log odds ratio**

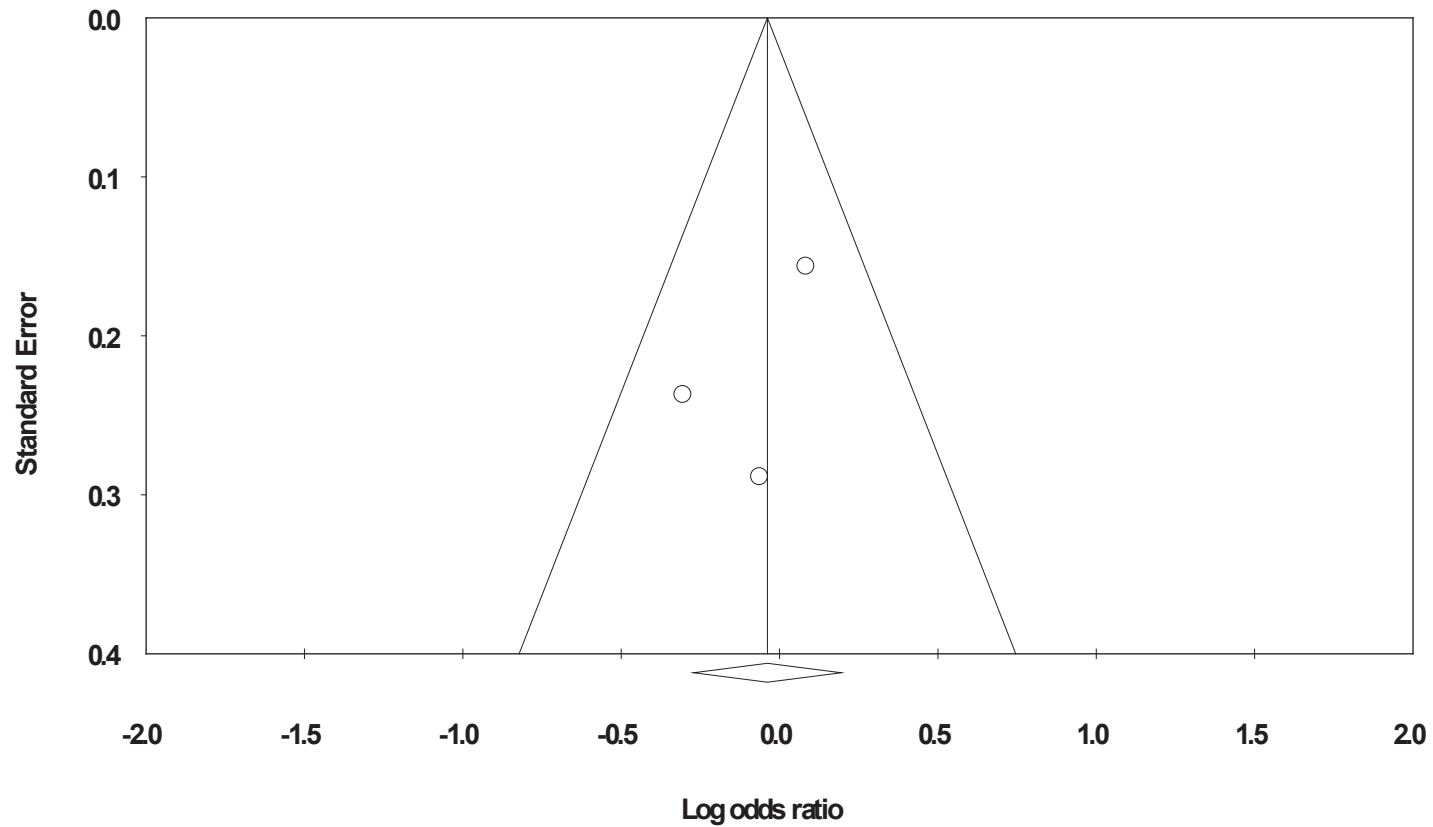

Supplement: Additional file 2: — Funnel plots of nineteen SNPs with more than two studies. a: Funnel plot of ADIPOQ rs17300539. b: Funnel plot of CCR5 rs333. c: Funnel plot of CCR5 rs2734648. d: Funnel plot of EPO rs1617640. e: Funnel plot of CCR5 rs1799987. f: Funnel plot of RANTES rs2107538. g: Funnel plot of TGF-B1 rs1800468. h: Funnel plot of TGF-B1 rs1800469. i: Funnel plot of TGF-B1 rs2241717. j: Funnel plot of TGF-B1 rs8179181. k: Funnel plot of TGF-BR1 rs928180. l: Funnel plot of TGF-BR1 rs1571589. m: Funnel plot of TGF-B1 rs1800471. n: Funnel plot of TGF-B1 rs1800470. o: Funnel plot of VEGFA rs3024997. p: Funnel plot of VEGFA rs3025000. q: Funnel plot of VEGFA rs2146323. r: Funnel plot of VEGFB rs12366035. s: Funnel plot of VEGFC rs585706. [file 12881_2014_103_MOESM2_ESM.zip › Additional file 2/7053225111232618_add2h.pdf]

**Funnel Plot of Standard Error by Log odds ratio**

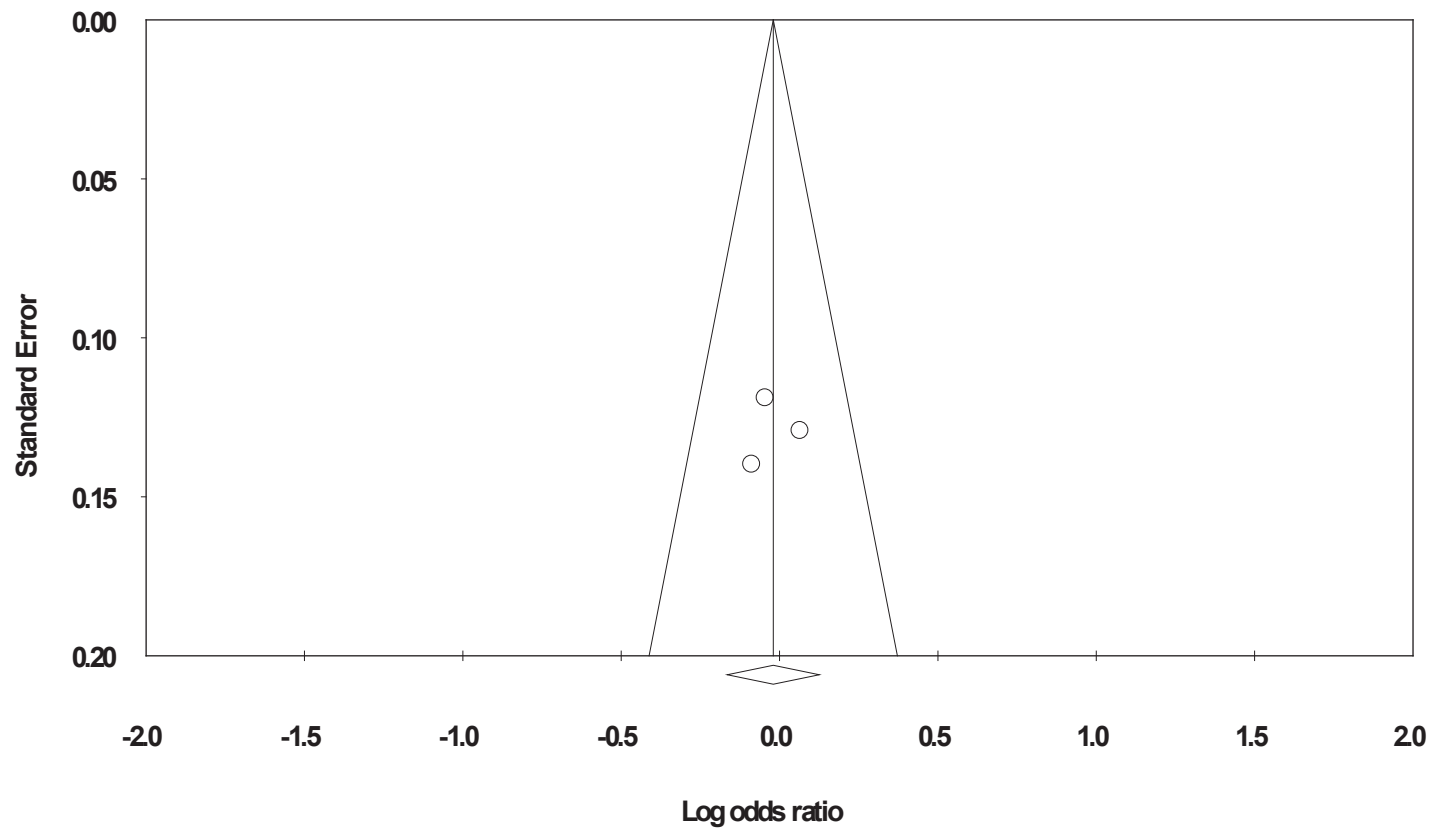

Supplement: Additional file 2: — Funnel plots of nineteen SNPs with more than two studies. a: Funnel plot of ADIPOQ rs17300539. b: Funnel plot of CCR5 rs333. c: Funnel plot of CCR5 rs2734648. d: Funnel plot of EPO rs1617640. e: Funnel plot of CCR5 rs1799987. f: Funnel plot of RANTES rs2107538. g: Funnel plot of TGF-B1 rs1800468. h: Funnel plot of TGF-B1 rs1800469. i: Funnel plot of TGF-B1 rs2241717. j: Funnel plot of TGF-B1 rs8179181. k: Funnel plot of TGF-BR1 rs928180. l: Funnel plot of TGF-BR1 rs1571589. m: Funnel plot of TGF-B1 rs1800471. n: Funnel plot of TGF-B1 rs1800470. o: Funnel plot of VEGFA rs3024997. p: Funnel plot of VEGFA rs3025000. q: Funnel plot of VEGFA rs2146323. r: Funnel plot of VEGFB rs12366035. s: Funnel plot of VEGFC rs585706. [file 12881_2014_103_MOESM2_ESM.zip › Additional file 2/7053225111232618_add2i.pdf]

**Funnel Plot of Standard Error by Log odds ratio**

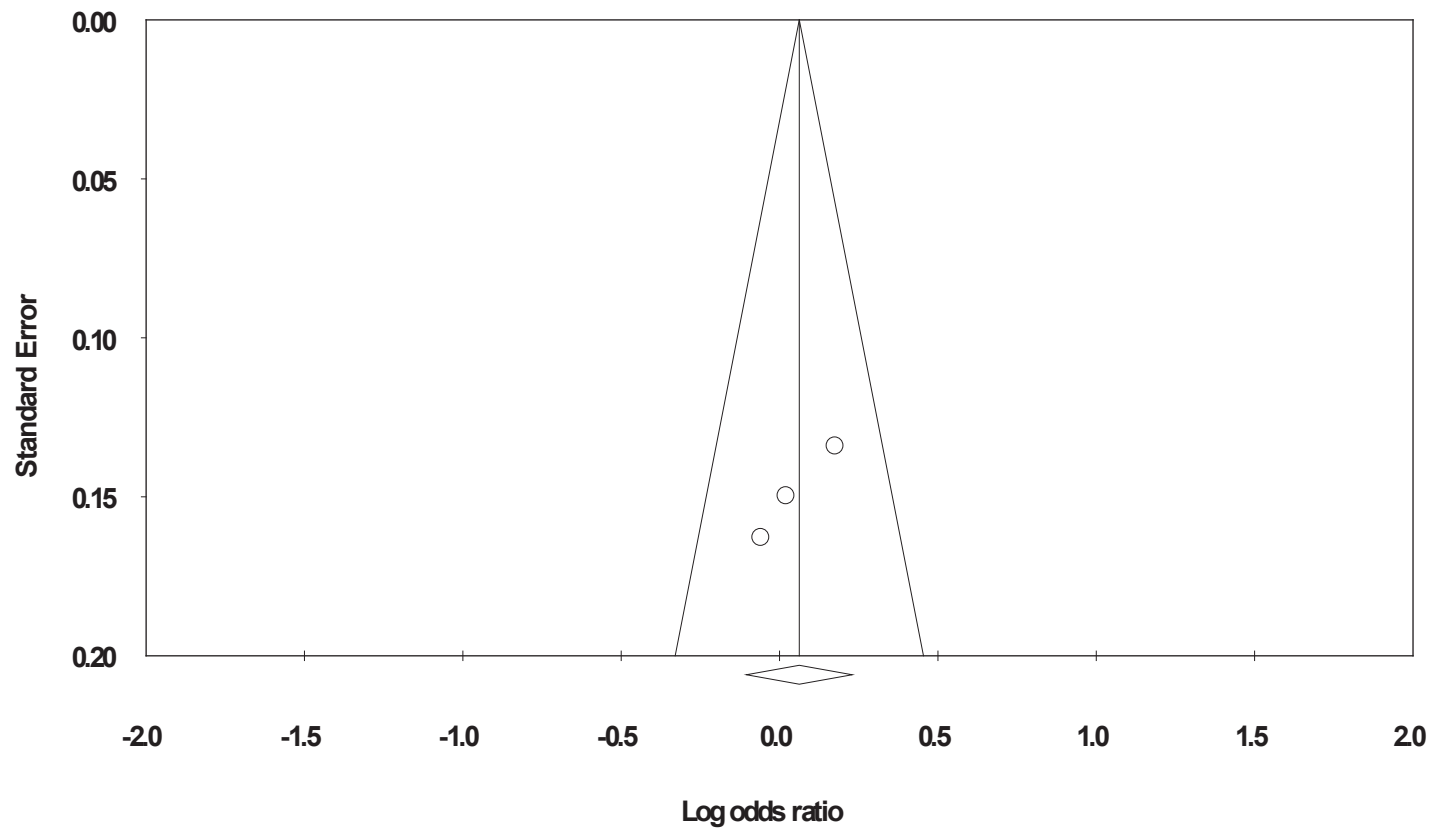

Supplement: Additional file 2: — Funnel plots of nineteen SNPs with more than two studies. a: Funnel plot of ADIPOQ rs17300539. b: Funnel plot of CCR5 rs333. c: Funnel plot of CCR5 rs2734648. d: Funnel plot of EPO rs1617640. e: Funnel plot of CCR5 rs1799987. f: Funnel plot of RANTES rs2107538. g: Funnel plot of TGF-B1 rs1800468. h: Funnel plot of TGF-B1 rs1800469. i: Funnel plot of TGF-B1 rs2241717. j: Funnel plot of TGF-B1 rs8179181. k: Funnel plot of TGF-BR1 rs928180. l: Funnel plot of TGF-BR1 rs1571589. m: Funnel plot of TGF-B1 rs1800471. n: Funnel plot of TGF-B1 rs1800470. o: Funnel plot of VEGFA rs3024997. p: Funnel plot of VEGFA rs3025000. q: Funnel plot of VEGFA rs2146323. r: Funnel plot of VEGFB rs12366035. s: Funnel plot of VEGFC rs585706. [file 12881_2014_103_MOESM2_ESM.zip › Additional file 2/7053225111232618_add2j.pdf]

**Funnel Plot of Standard Error by Log odds ratio**

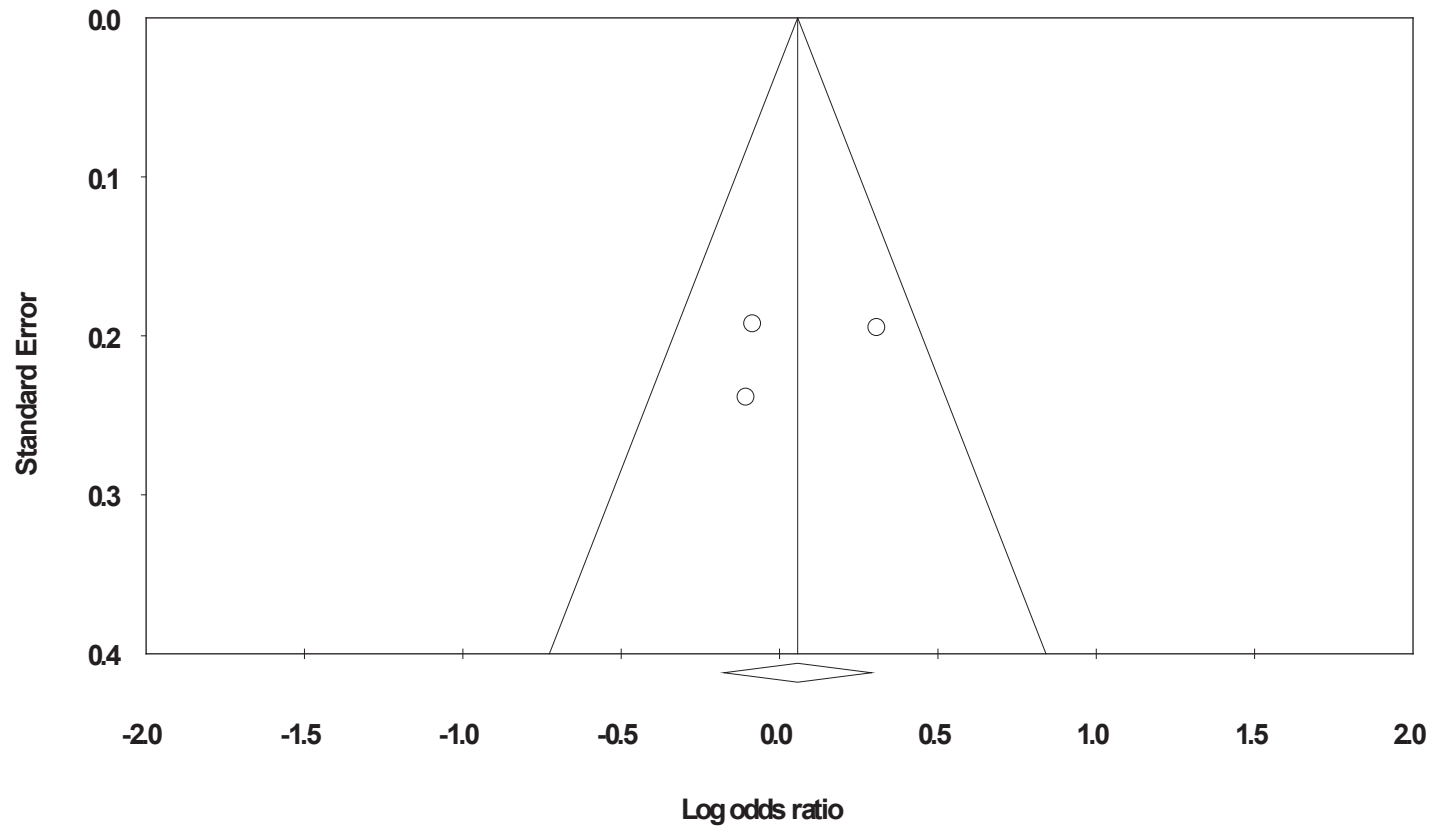

Supplement: Additional file 2: — Funnel plots of nineteen SNPs with more than two studies. a: Funnel plot of ADIPOQ rs17300539. b: Funnel plot of CCR5 rs333. c: Funnel plot of CCR5 rs2734648. d: Funnel plot of EPO rs1617640. e: Funnel plot of CCR5 rs1799987. f: Funnel plot of RANTES rs2107538. g: Funnel plot of TGF-B1 rs1800468. h: Funnel plot of TGF-B1 rs1800469. i: Funnel plot of TGF-B1 rs2241717. j: Funnel plot of TGF-B1 rs8179181. k: Funnel plot of TGF-BR1 rs928180. l: Funnel plot of TGF-BR1 rs1571589. m: Funnel plot of TGF-B1 rs1800471. n: Funnel plot of TGF-B1 rs1800470. o: Funnel plot of VEGFA rs3024997. p: Funnel plot of VEGFA rs3025000. q: Funnel plot of VEGFA rs2146323. r: Funnel plot of VEGFB rs12366035. s: Funnel plot of VEGFC rs585706. [file 12881_2014_103_MOESM2_ESM.zip › Additional file 2/7053225111232618_add2k.pdf]

**Funnel Plot of Standard Error by Log odds ratio**

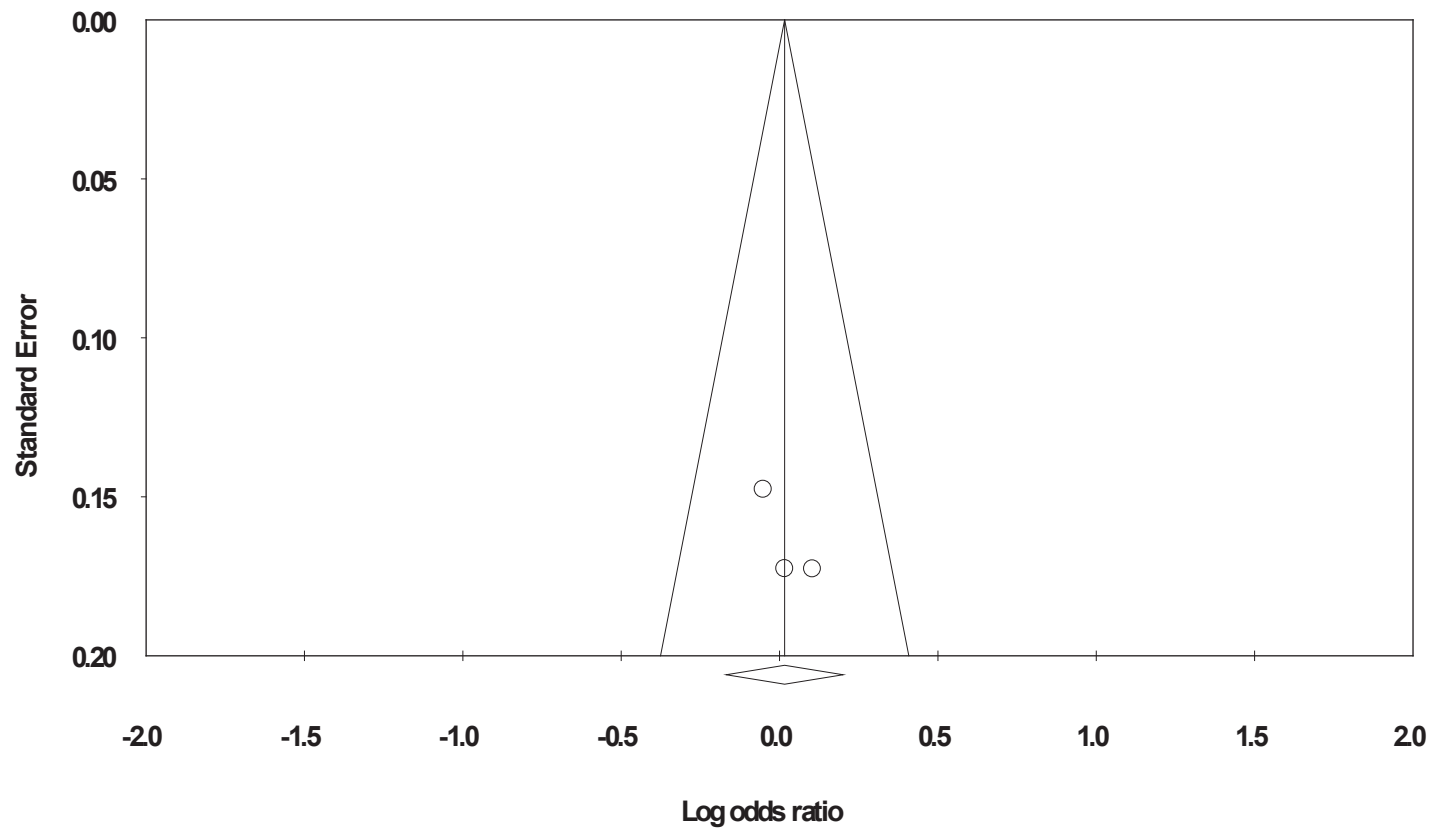

Supplement: Additional file 2: — Funnel plots of nineteen SNPs with more than two studies. a: Funnel plot of ADIPOQ rs17300539. b: Funnel plot of CCR5 rs333. c: Funnel plot of CCR5 rs2734648. d: Funnel plot of EPO rs1617640. e: Funnel plot of CCR5 rs1799987. f: Funnel plot of RANTES rs2107538. g: Funnel plot of TGF-B1 rs1800468. h: Funnel plot of TGF-B1 rs1800469. i: Funnel plot of TGF-B1 rs2241717. j: Funnel plot of TGF-B1 rs8179181. k: Funnel plot of TGF-BR1 rs928180. l: Funnel plot of TGF-BR1 rs1571589. m: Funnel plot of TGF-B1 rs1800471. n: Funnel plot of TGF-B1 rs1800470. o: Funnel plot of VEGFA rs3024997. p: Funnel plot of VEGFA rs3025000. q: Funnel plot of VEGFA rs2146323. r: Funnel plot of VEGFB rs12366035. s: Funnel plot of VEGFC rs585706. [file 12881_2014_103_MOESM2_ESM.zip › Additional file 2/7053225111232618_add2l.pdf]

**Funnel Plot of Standard Error by Log odds ratio**

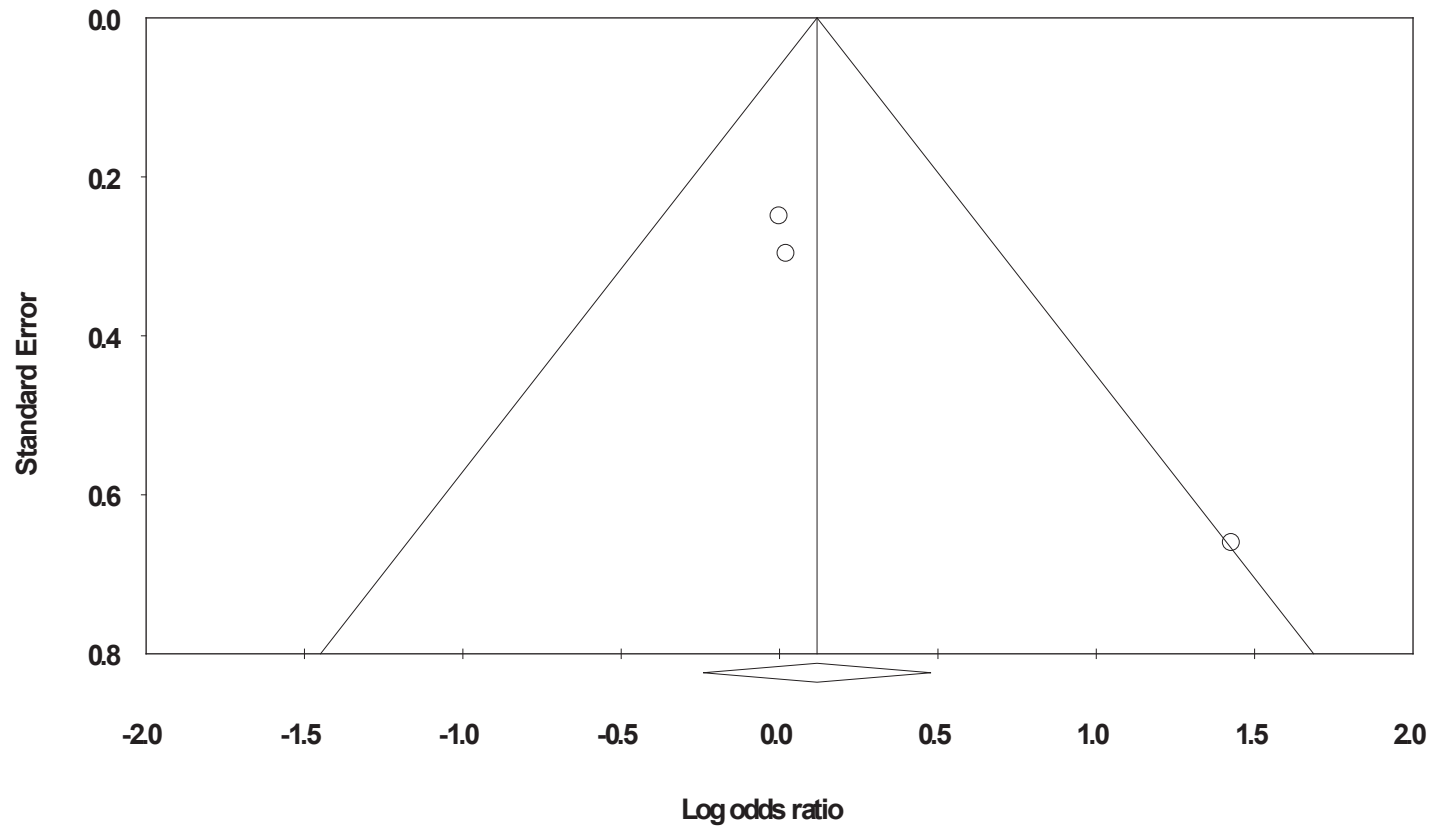

Supplement: Additional file 2: — Funnel plots of nineteen SNPs with more than two studies. a: Funnel plot of ADIPOQ rs17300539. b: Funnel plot of CCR5 rs333. c: Funnel plot of CCR5 rs2734648. d: Funnel plot of EPO rs1617640. e: Funnel plot of CCR5 rs1799987. f: Funnel plot of RANTES rs2107538. g: Funnel plot of TGF-B1 rs1800468. h: Funnel plot of TGF-B1 rs1800469. i: Funnel plot of TGF-B1 rs2241717. j: Funnel plot of TGF-B1 rs8179181. k: Funnel plot of TGF-BR1 rs928180. l: Funnel plot of TGF-BR1 rs1571589. m: Funnel plot of TGF-B1 rs1800471. n: Funnel plot of TGF-B1 rs1800470. o: Funnel plot of VEGFA rs3024997. p: Funnel plot of VEGFA rs3025000. q: Funnel plot of VEGFA rs2146323. r: Funnel plot of VEGFB rs12366035. s: Funnel plot of VEGFC rs585706. [file 12881_2014_103_MOESM2_ESM.zip › Additional file 2/7053225111232618_add2m.pdf]

**Funnel Plot of Standard Error by Log odds ratio**

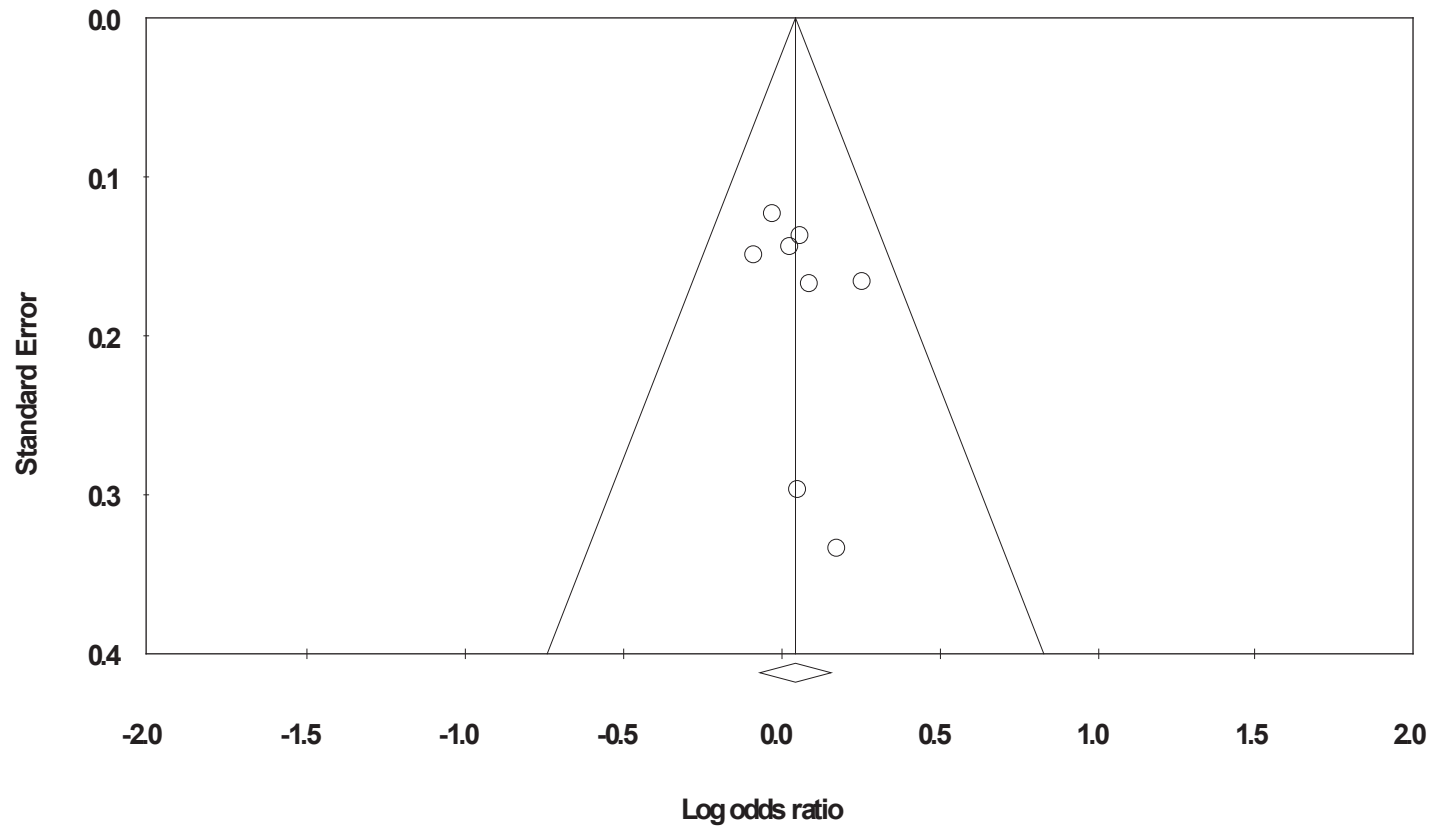

Supplement: Additional file 2: — Funnel plots of nineteen SNPs with more than two studies. a: Funnel plot of ADIPOQ rs17300539. b: Funnel plot of CCR5 rs333. c: Funnel plot of CCR5 rs2734648. d: Funnel plot of EPO rs1617640. e: Funnel plot of CCR5 rs1799987. f: Funnel plot of RANTES rs2107538. g: Funnel plot of TGF-B1 rs1800468. h: Funnel plot of TGF-B1 rs1800469. i: Funnel plot of TGF-B1 rs2241717. j: Funnel plot of TGF-B1 rs8179181. k: Funnel plot of TGF-BR1 rs928180. l: Funnel plot of TGF-BR1 rs1571589. m: Funnel plot of TGF-B1 rs1800471. n: Funnel plot of TGF-B1 rs1800470. o: Funnel plot of VEGFA rs3024997. p: Funnel plot of VEGFA rs3025000. q: Funnel plot of VEGFA rs2146323. r: Funnel plot of VEGFB rs12366035. s: Funnel plot of VEGFC rs585706. [file 12881_2014_103_MOESM2_ESM.zip › Additional file 2/7053225111232618_add2n.pdf]

**Funnel Plot of Standard Error by Log odds ratio**

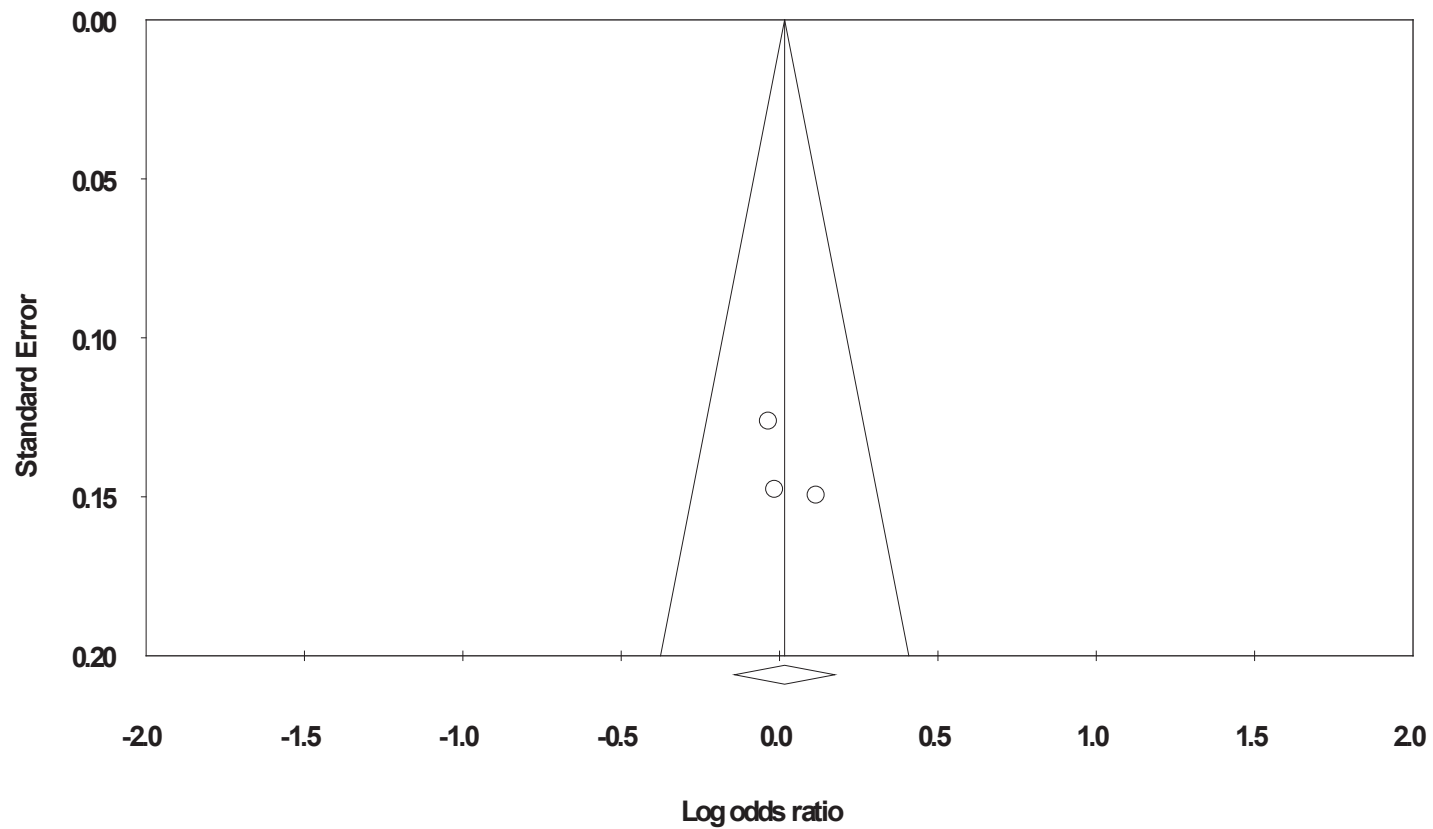

Supplement: Additional file 2: — Funnel plots of nineteen SNPs with more than two studies. a: Funnel plot of ADIPOQ rs17300539. b: Funnel plot of CCR5 rs333. c: Funnel plot of CCR5 rs2734648. d: Funnel plot of EPO rs1617640. e: Funnel plot of CCR5 rs1799987. f: Funnel plot of RANTES rs2107538. g: Funnel plot of TGF-B1 rs1800468. h: Funnel plot of TGF-B1 rs1800469. i: Funnel plot of TGF-B1 rs2241717. j: Funnel plot of TGF-B1 rs8179181. k: Funnel plot of TGF-BR1 rs928180. l: Funnel plot of TGF-BR1 rs1571589. m: Funnel plot of TGF-B1 rs1800471. n: Funnel plot of TGF-B1 rs1800470. o: Funnel plot of VEGFA rs3024997. p: Funnel plot of VEGFA rs3025000. q: Funnel plot of VEGFA rs2146323. r: Funnel plot of VEGFB rs12366035. s: Funnel plot of VEGFC rs585706. [file 12881_2014_103_MOESM2_ESM.zip › Additional file 2/7053225111232618_add2o.pdf]

**Funnel Plot of Standard Error by Log odds ratio**

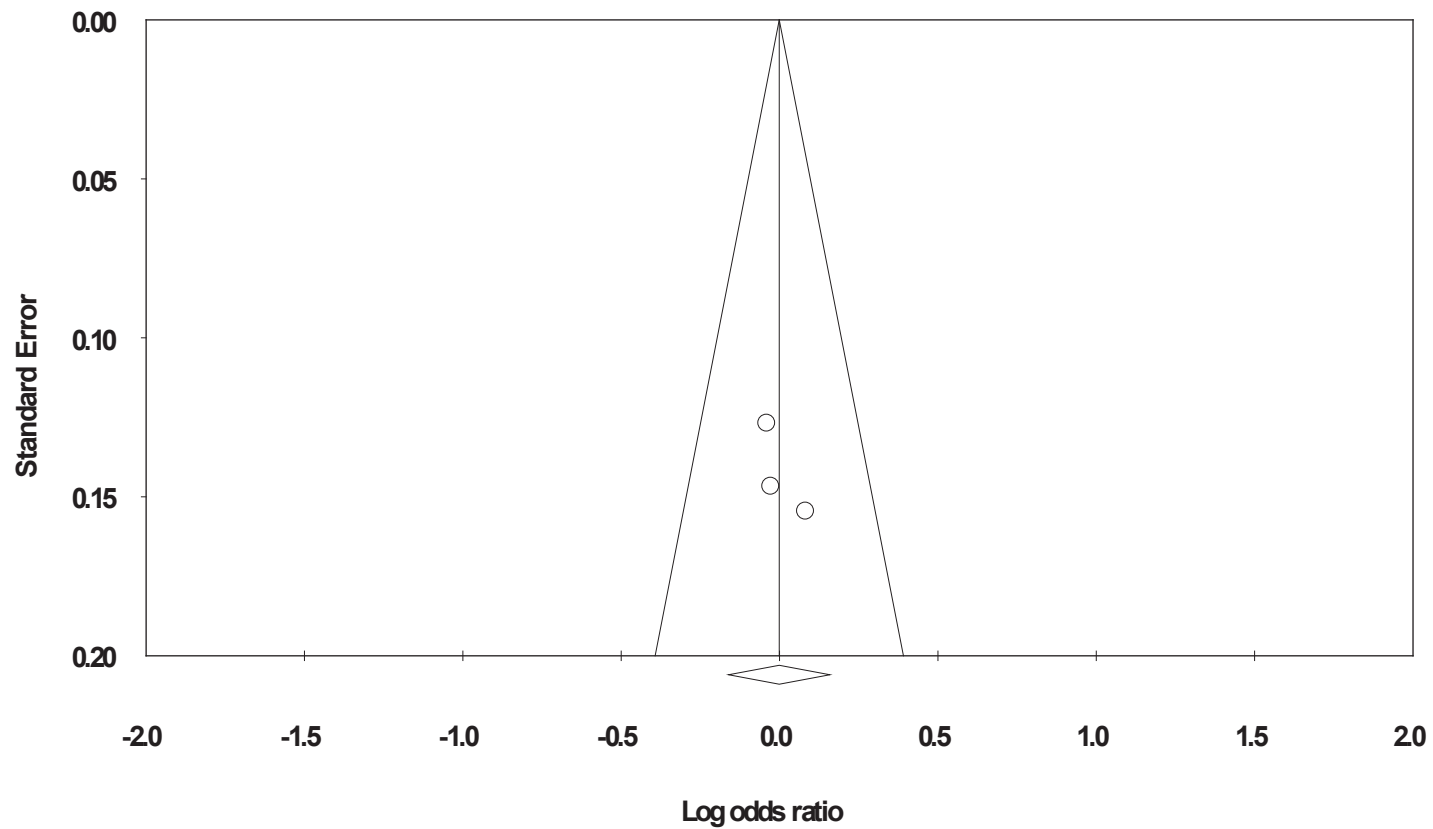

Supplement: Additional file 2: — Funnel plots of nineteen SNPs with more than two studies. a: Funnel plot of ADIPOQ rs17300539. b: Funnel plot of CCR5 rs333. c: Funnel plot of CCR5 rs2734648. d: Funnel plot of EPO rs1617640. e: Funnel plot of CCR5 rs1799987. f: Funnel plot of RANTES rs2107538. g: Funnel plot of TGF-B1 rs1800468. h: Funnel plot of TGF-B1 rs1800469. i: Funnel plot of TGF-B1 rs2241717. j: Funnel plot of TGF-B1 rs8179181. k: Funnel plot of TGF-BR1 rs928180. l: Funnel plot of TGF-BR1 rs1571589. m: Funnel plot of TGF-B1 rs1800471. n: Funnel plot of TGF-B1 rs1800470. o: Funnel plot of VEGFA rs3024997. p: Funnel plot of VEGFA rs3025000. q: Funnel plot of VEGFA rs2146323. r: Funnel plot of VEGFB rs12366035. s: Funnel plot of VEGFC rs585706. [file 12881_2014_103_MOESM2_ESM.zip › Additional file 2/7053225111232618_add2p.pdf]

**Funnel Plot of Standard Error by Log odds ratio**

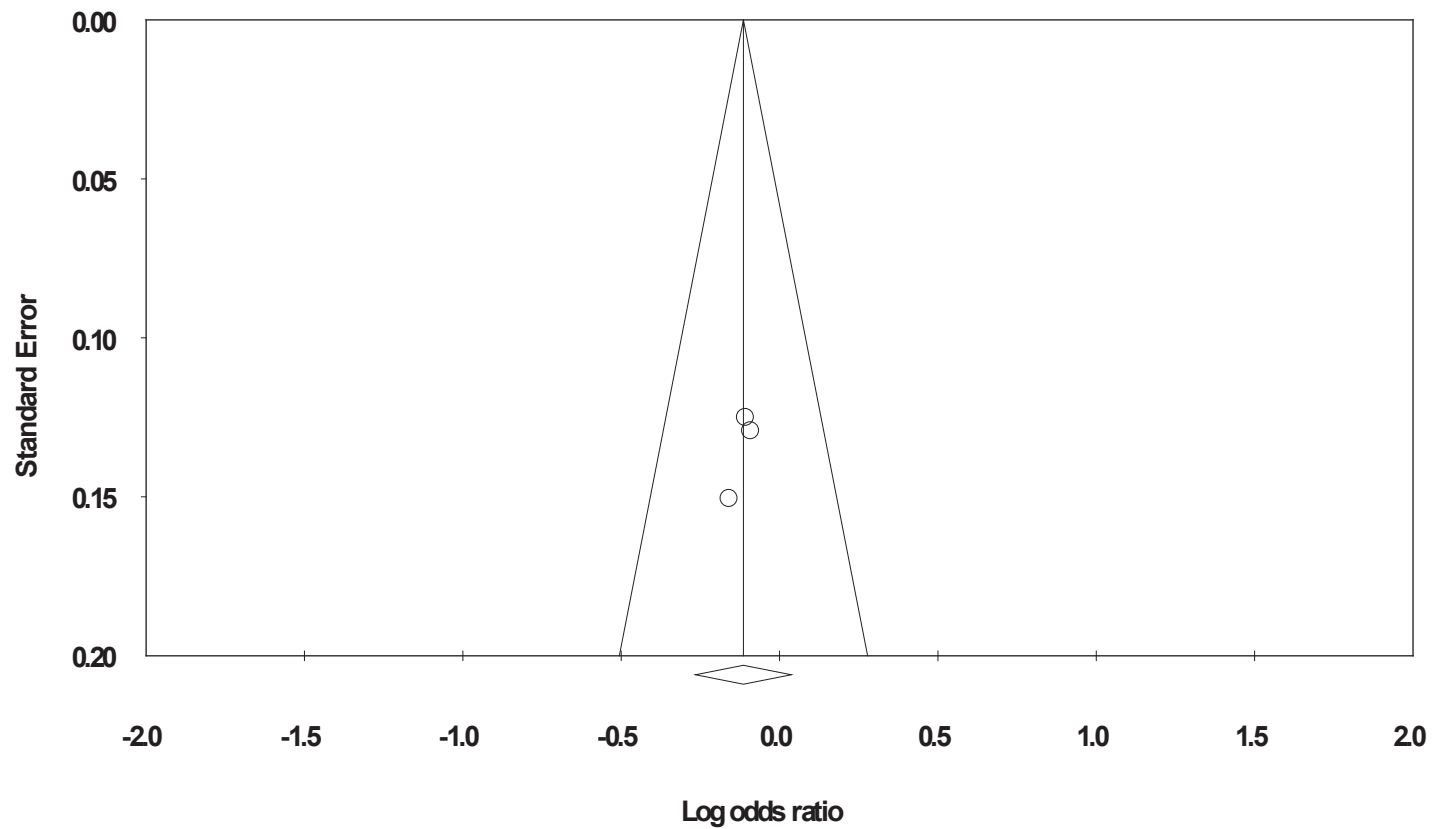

Supplement: Additional file 2: — Funnel plots of nineteen SNPs with more than two studies. a: Funnel plot of ADIPOQ rs17300539. b: Funnel plot of CCR5 rs333. c: Funnel plot of CCR5 rs2734648. d: Funnel plot of EPO rs1617640. e: Funnel plot of CCR5 rs1799987. f: Funnel plot of RANTES rs2107538. g: Funnel plot of TGF-B1 rs1800468. h: Funnel plot of TGF-B1 rs1800469. i: Funnel plot of TGF-B1 rs2241717. j: Funnel plot of TGF-B1 rs8179181. k: Funnel plot of TGF-BR1 rs928180. l: Funnel plot of TGF-BR1 rs1571589. m: Funnel plot of TGF-B1 rs1800471. n: Funnel plot of TGF-B1 rs1800470. o: Funnel plot of VEGFA rs3024997. p: Funnel plot of VEGFA rs3025000. q: Funnel plot of VEGFA rs2146323. r: Funnel plot of VEGFB rs12366035. s: Funnel plot of VEGFC rs585706. [file 12881_2014_103_MOESM2_ESM.zip › Additional file 2/7053225111232618_add2q.pdf]

**Funnel Plot of Standard Error by Log odds ratio**

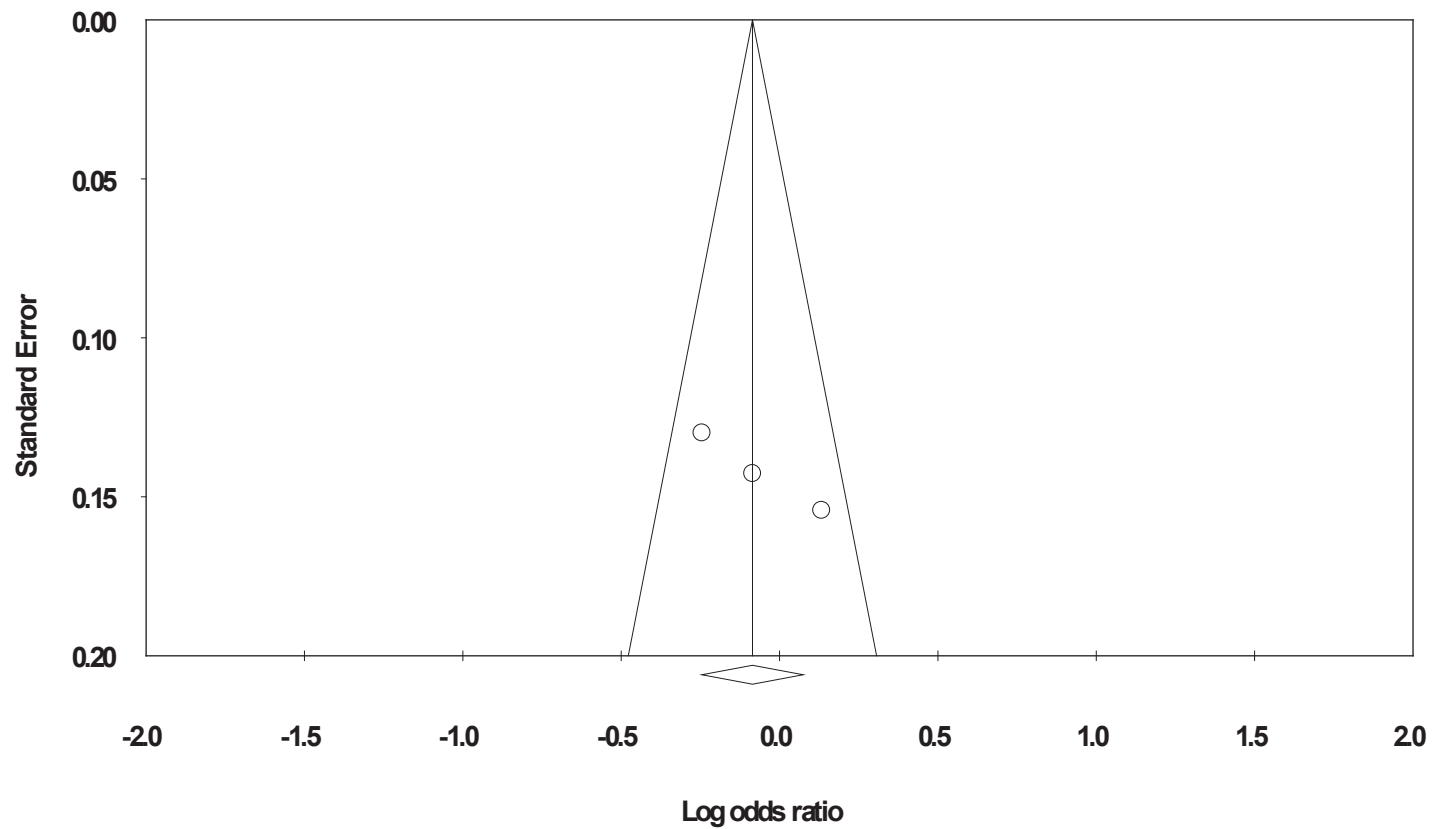

Supplement: Additional file 2: — Funnel plots of nineteen SNPs with more than two studies. a: Funnel plot of ADIPOQ rs17300539. b: Funnel plot of CCR5 rs333. c: Funnel plot of CCR5 rs2734648. d: Funnel plot of EPO rs1617640. e: Funnel plot of CCR5 rs1799987. f: Funnel plot of RANTES rs2107538. g: Funnel plot of TGF-B1 rs1800468. h: Funnel plot of TGF-B1 rs1800469. i: Funnel plot of TGF-B1 rs2241717. j: Funnel plot of TGF-B1 rs8179181. k: Funnel plot of TGF-BR1 rs928180. l: Funnel plot of TGF-BR1 rs1571589. m: Funnel plot of TGF-B1 rs1800471. n: Funnel plot of TGF-B1 rs1800470. o: Funnel plot of VEGFA rs3024997. p: Funnel plot of VEGFA rs3025000. q: Funnel plot of VEGFA rs2146323. r: Funnel plot of VEGFB rs12366035. s: Funnel plot of VEGFC rs585706. [file 12881_2014_103_MOESM2_ESM.zip › Additional file 2/7053225111232618_add2r.pdf]

**Funnel Plot of Standard Error by Log odds ratio**

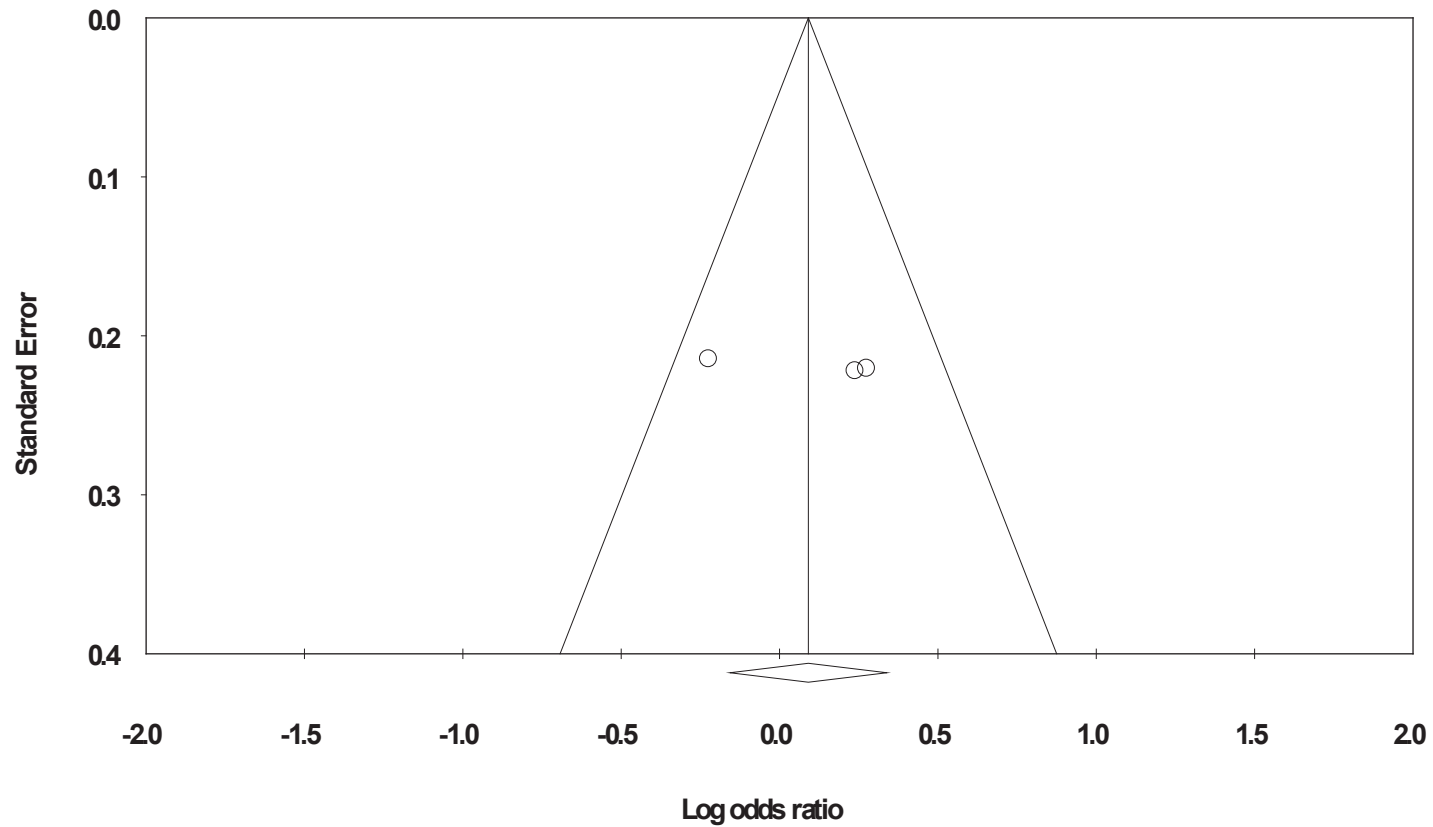

Supplement: Additional file 2: — Funnel plots of nineteen SNPs with more than two studies. a: Funnel plot of ADIPOQ rs17300539. b: Funnel plot of CCR5 rs333. c: Funnel plot of CCR5 rs2734648. d: Funnel plot of EPO rs1617640. e: Funnel plot of CCR5 rs1799987. f: Funnel plot of RANTES rs2107538. g: Funnel plot of TGF-B1 rs1800468. h: Funnel plot of TGF-B1 rs1800469. i: Funnel plot of TGF-B1 rs2241717. j: Funnel plot of TGF-B1 rs8179181. k: Funnel plot of TGF-BR1 rs928180. l: Funnel plot of TGF-BR1 rs1571589. m: Funnel plot of TGF-B1 rs1800471. n: Funnel plot of TGF-B1 rs1800470. o: Funnel plot of VEGFA rs3024997. p: Funnel plot of VEGFA rs3025000. q: Funnel plot of VEGFA rs2146323. r: Funnel plot of VEGFB rs12366035. s: Funnel plot of VEGFC rs585706. [file 12881_2014_103_MOESM2_ESM.zip › Additional file 2/7053225111232618_add2s.pdf]
